# RNA Sequencing Reveals that Kaposi Sarcoma-Associated Herpesvirus Infection Mimics Hypoxia Gene Expression Signature

**DOI:** 10.1371/journal.ppat.1006143

**Published:** 2017-01-03

**Authors:** Coralie Viollet, David A. Davis, Shewit S. Tekeste, Martin Reczko, Joseph M. Ziegelbauer, Francesco Pezzella, Jiannis Ragoussis, Robert Yarchoan

**Affiliations:** 1 HIV and AIDS Malignancy Branch, Center for Cancer Research, National Cancer Institute, National Institutes of Health, Bethesda, Maryland, United States of America; 2 The Wellcome Trust Centre for Human Genetics, University of Oxford, Oxford, United Kingdom; 3 Institute of Molecular Oncology, Alexander Fleming Biomedical Sciences Research Center, Vari, Greece; 4 Nuffield Division of Clinical Laboratory Sciences, University of Oxford, Oxford, United Kingdom; 5 McGill University and Génome Québec Innovation Centre, Montréal, Québec, Canada; 6 Department of Biochemistry, King Abdulaziz University, Jeddah, Saudi Arabia; Wistar Institute, UNITED STATES

## Abstract

Kaposi sarcoma-associated herpesvirus (KSHV) causes several tumors and hyperproliferative disorders. Hypoxia and hypoxia-inducible factors (HIFs) activate latent and lytic KSHV genes, and several KSHV proteins increase the cellular levels of HIF. Here, we used RNA sequencing, qRT-PCR, Taqman assays, and pathway analysis to explore the miRNA and mRNA response of uninfected and KSHV-infected cells to hypoxia, to compare this with the genetic changes seen in chronic latent KSHV infection, and to explore the degree to which hypoxia and KSHV infection interact in modulating mRNA and miRNA expression. We found that the gene expression signatures for KSHV infection and hypoxia have a 34% overlap. Moreover, there were considerable similarities between the genes up-regulated by hypoxia in uninfected (SLK) and in KSHV-infected (SLKK) cells. hsa-miR-210, a HIF-target known to have pro-angiogenic and anti-apoptotic properties, was significantly up-regulated by both KSHV infection and hypoxia using Taqman assays. Interestingly, expression of KSHV-encoded miRNAs was not affected by hypoxia. These results demonstrate that KSHV harnesses a part of the hypoxic cellular response and that a substantial portion of hypoxia-induced changes in cellular gene expression are induced by KSHV infection. Therefore, targeting hypoxic pathways may be a useful way to develop therapeutic strategies for KSHV-related diseases.

## Author Summary

Kaposi sarcoma-associated herpesvirus (KSHV) is an oncogenic herpesvirus known to cause several tumors and hyperproliferative disorders. While there has been reports of KSHV activating and increasing hypoxia-inducible factors (HIFs), this is the first report investigating and establishing the extent to which KSHV has evolved to reproduce the effects of hypoxia. We demonstrate that the cellular changes in gene expression induced by KSHV infection include many of the changes induced by hypoxia. This has substantial implications for the biology of KSHV and the pathogenesis of KSHV-associated cancers. To achieve this, we used mRNA-sequencing and small RNA-sequencing in combination with bioinformatics analysis, and orthogonal assays such as qRT-PCR and Taqman assays to determine the effects of hypoxia on miRNA and mRNA expression. We showed that not only was there a 34% overlap between the hypoxic response and KSHV infection, but also that miRNA miR-210, a HIF-target known to have anti-apoptotic, angiogenic, and oncogenic properties, was independently and additively increased by KSHV infection and hypoxia. Furthermore, we explored the effects of hypoxia on KSHV miRNAs and consistently observed that none of the KSHV miRNAs are affected by oxygen deprivation. These studies suggest that KSHV harnesses a part of the hypoxic cellular response and that a substantial portion of hypoxia-induced changes in cellular gene expression are induced by KSHV infection. Previous studies have shown that hypoxia and HIFs activate KSHV-encoded genes, including several involved in tumor formation. These findings suggest that targeting HIFs or hypoxia pathways could block this positive feedback loop between KSHV and hypoxia and thus might be a useful strategy to treat KSHV-related tumors or other diseases. We believe these are important findings with broad implication for the understanding of the biology of KSHV and other oncoviruses and the pathogenesis of KSHV-induced tumors. As such, it should be of interest to the broad community of investigators and clinicians interested in the biology of oncoviruses, virus-induced cellular changes, and the pathogenesis and therapy of virus-induced tumors.

## Introduction

Kaposi sarcoma-associated herpesvirus (KSHV) is the etiologic agent for several hyperproliferative disorders and tumors, including Kaposi’s sarcoma (KS), primary effusion lymphoma (PEL) and a form of multicentric Castleman disease (MCD) [1–4]. Like other herpesviruses, KSHV has two patterns of gene expression: latent, in which only a small subset of genes are expressed; and lytic, in which the full repertoire of genes are expressed and viral progeny are produced [5]. A number of recent studies have shown that hypoxia and hypoxia-inducible factors (HIFs) are important in the KSHV life cycle and the pathogenesis of KSHV-induced diseases [6–8]. Two of the tumors caused by KSHV, KS and PEL, preferentially arise in relatively hypoxic environments: the extremities and pleural effusions, respectively [9,10]. Cells respond to hypoxic environments by a rapid up-regulation in their levels of two main HIFs, HIF-1 and HIF-2, which in turn enter the nucleus and activate HIF-responsive genes by binding to hypoxia response elements (HRE) in their promoter regions [11,12]. Hypoxia and HIFs can also up-regulate levels of the cellular microRNA (miRNA), miR-210, which in turn affects a number of target genes to promote adaptation to hypoxia [13,14].

Interestingly, exposure of KSHV-infected PEL cells to hypoxia or to HIFs has been shown to induce lytic KSHV replication [7] and also to directly up-regulate certain KSHV genes, including the lytic switch gene replication transcription activator (RTA) [6], the open reading frame (ORF) 34 to 37 cluster of lytic genes [15], and latency-associated nuclear antigen (LANA) [16].

KSHV infection can induce a number of changes in the gene expression pattern of target cells that facilitate viral infection, persistence, avoidance of host antiviral strategies, and when appropriate, viral replication. Some of these changes are induced by KSHV proteins, which can modulate a number of intracellular signaling pathways and the production of growth factors and cytokines [17,18]. Other changes are caused by KSHV-encoded miRNAs, which are generated from a dozen precursor miRNAs [19–21]. It has been shown that among the changes mediated by KSHV are the induction of a hypoxic phenotype and the increase in the levels of and activation of HIFs under certain conditions [16,22,23]. These changes are mediated by several KSHV-encoded proteins, including latency-associated nuclear antigen (LANA) [16], viral interferon regulatory factor 3 (v-IRF3) [24], and viral G protein-coupled receptor (v-GPCR) [22]. However, whether or not these three KSHV proteins are necessary to activate HIF is unknown.

Our group recently used next-generation sequencing (NGS) analysis to explore the changes in cellular mRNA and miRNA expression profiles induced by latent KSHV infection using the SLK tumor line [25]. Under normoxic conditions, KSHV infection modulated many mRNAs including a number that had been previously reported to be affected by hypoxia. Also, expression of a number of human miRNAs was different in KSHV-infected SLKK cells as compared to uninfected SLK cells, including a small but statistically significant up-regulation of miR-210 [25]. In the current study, we extended these findings by using NGS to assess the changes in mRNA and miRNA expression profiles induced in this same SLK line by exposure to hypoxia, and we compared the findings to those induced by chronic KSHV infection. In addition, we studied the changes in mRNA and miRNA expression profiles after exposure to hypoxia in SLKK cells to those in KSHV-uninfected SLK cells in order to understand how hypoxia and KSHV may interact to modulate cellular gene expression.

## Results

### Cellular mRNA response to hypoxia in uninfected SLK cells

**Fig 1:**
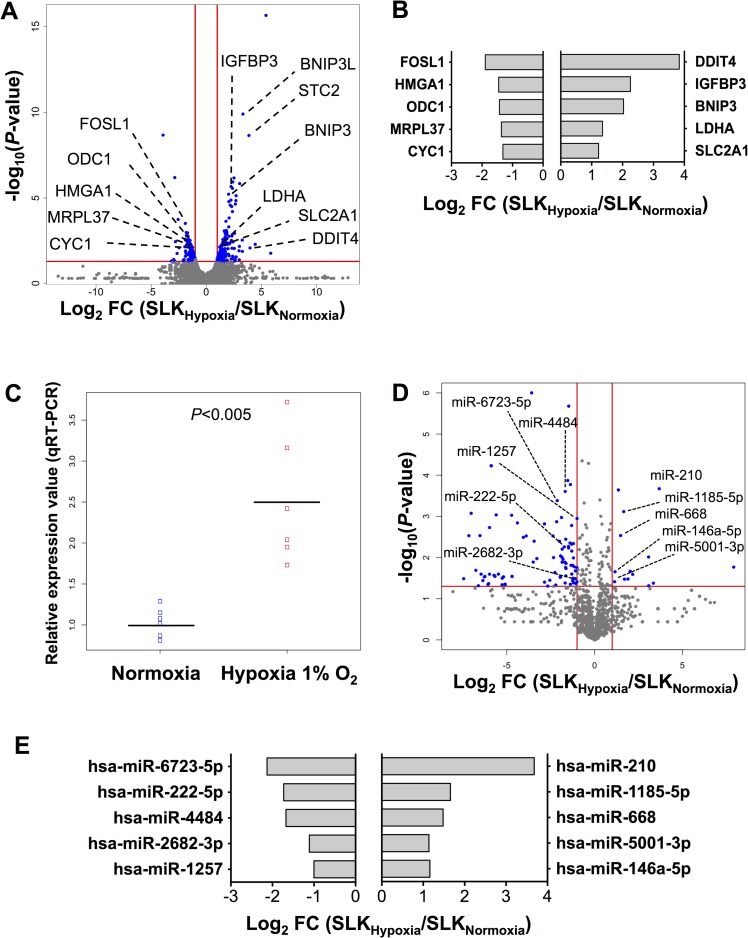
Differential expression of mRNAs and miRNAs in hypoxic vs. normoxic uninfected SLK cells. Uninfected SLK cells were incubated under standard (normoxic, 21% O_2_) conditions or in hypoxia (1% O_2_) (n = 3). After 24hrs, total RNA was extracted. cDNA libraries for small RNA and mRNA were prepared and sequenced on the Illumina HiSeq platform (Material and Methods). (**A**) Volcano plot of mRNA differential expression in hypoxic vs. normoxic uninfected SLK cells. Vertical lines indicate the threshold for a relative expression fold change (FC) of 2 or -2 compared to normoxic controls. The horizontal red line represents the threshold of a 0.05 *P*-value. The blue dots lying in the top right and top left sectors represent significantly up-and down-regulated mRNAs, respectively, in hypoxic vs. normoxic SLK cells (*P* ≤0.05, linear FC ≤-2 or ≥2). Only annotated genes are shown in this volcano plot. (**B**) Top 5 most abundant mRNAs up-regulated and down-regulated in hypoxic vs. normoxic uninfected SLK cells. Bars left and right of the y-axis show the 5 most abundant mRNAs that are repressed and induced, respectively, in hypoxia compared to normoxia in SLK cells. The mRNAs were ordered by fold change. In this graph, the *P*-value threshold was set at ≤0.01 to limit the analysis to genes whose modulation by hypoxia was highly significant. (**C**) VEGF differential expression in hypoxic vs. normoxic SLK cells. This plot represents the fold difference in VEGF expression between hypoxia and normoxia in SLK cells, measured by qRT-PCR assays (n = 6). Ribosomal gene 18S was used for internal normalisation. Hypoxic samples were normalised to normoxic samples (n = 6). (**D**) Volcano plot of miRNA differential expression in hypoxic and normoxic uninfected SLK cells. The plot is depicted as in **Fig 1A**, with vertical and horizontal lines indicating the threshold for a relative expression fold change of 2 or -2 compared to normoxic controls, and the threshold of a 0.05 *P*-value, respectively. (**E**) Top 5 most abundant miRNAs up-regulated and down-regulated in hypoxic vs. normoxic uninfected SLK cells. Bars left and right of the y-axis show the 5 most abundant miRNAs that are repressed and induced, respectively, in hypoxia compared to normoxia in SLK cells (*P*≤0.05).

To assess the changes in mRNA gene expression induced by hypoxia in KSHV-uninfected SLK cells, next-generation sequencing was performed on mRNA libraries from hypoxic and normoxic uninfected SLK samples in triplicate. Out of a total of 30,808 mRNAs analyzed, 519 annotated genes were significantly differentially expressed (*P* ≤0.05, fold change ≤-2 or ≥2) in hypoxic vs. normoxic SLK cells (**Fig 1A**). The most abundant genes (average read count > 100) that were differentially expressed are depicted in **Fig 1B**. Importantly, among the 210 up-regulated genes were a number that have been described previously to be increased in hypoxia, such as BNIP3, BNIP3L, DDIT4, LDHA, SLC2A1 (GLUT1) and STC2 (**Fig 1A and 1B**) [26–31]. As a confirmation that expected cellular mRNAs were responding to hypoxia, we assessed the expression of vascular endothelial growth factor (VEGF), a well-described hypoxia-responsive gene [32], in normoxic and hypoxic SLK cells. By RNA-seq, VEGF expression was up-regulated 2.1 fold but that change was not considered significant as *P* = 0.06, which is above our threshold of *P*≤0.05. However, we separately confirmed by RT-qPCR that VEGF was significantly up-regulated ~2.5 fold by hypoxia (*P*<0.005, **Fig 1C**). Taken together, these results provide confidence that changes seen in the SLK cells under hypoxic treatment were in fact due to hypoxia.

### Cellular miRNA response to hypoxia in uninfected SLK cells

The same SLK hypoxic samples that were analyzed for mRNA-Seq were also sequenced for small RNA-Seq to assess differential miRNA expression. A total of 112 out of 1,378 miRNAs were found significantly deregulated in hypoxia compared to normoxia (*P* ≤0.05, FC ≤-2 or ≥2; **Fig 1D**, top left and right sectors). Perhaps the most striking observation was that of the 112 miRNAs deregulated in response to hypoxia, a large majority (99 miRNAs, or 88%) were down-regulated. Of the 112 hypoxia-regulated miRNAs, also known as hypoxamiRs [33], 47 were expressed with an average miR read count greater than 1, illustrated by our heat map analysis; this showed a clear distinction between the triplicate hypoxic and normoxic samples (**S1A Fig**). Ten (21%) of the 47 hypoxamiRs with a read count greater than 1 were up-regulated, while 37 (79%) were down-regulated by hypoxia.

After a ranking based on read abundance, we determined the top 5 most abundant up- and down-regulated hypoxamiR in SLK cells (**Fig 1E**). As previously described for other cell types [34–36], of the most abundantly expressed miRNAs, miR-210 was the most significantly up-regulated by hypoxia in SLK cells with a nearly 13-fold increase (**Fig 1E**). Interestingly, we also observed an 8-fold increase of miR-210 host gene (MIR210HG), the precursor to miR-210, by RNA-Seq. This is consistent with previous findings showing that at least one functional HIF-1 binding site (hypoxia-response element, HRE) is located in the promoter region of miR-210 host gene [37]. A previously described hypoxamiR, miR-146a-5p, was also induced by hypoxia in SLK cells (2-fold), and has been shown to be a NF-κB-dependent gene which leads to the down-regulation of the inflammatory response [38]. MiRNAs miR-1185-5p, miR-668, and miR-5001-3p were all up-regulated (2 to 4 fold). On the other hand, miR-6723-5p, miR-222-5p, miR-4484, miR-2682-3p, and miR-1257 were the most down-regulated miRNAs (**Fig 1E**). Our small RNA-Seq study of uninfected SLK cells exposed to hypoxia confirmed previously published findings [37,38] and allowed for a global view of hypoxia-regulated miRNAs that will be compared to the effects of KSHV infection.

### Analysis of miRNA and mRNA target expression in hypoxic SLK cells

Ingenuity Pathway Analysis (IPA) was used to analyze the large number of deregulated genes in hypoxic SLK cells, in order to identify the principal pathways altered by hypoxia (**S2A Fig**). Among the top 15 pathways most significantly modulated by hypoxia were the Wnt/β-catenin signaling, the neuregulin signaling, prostanoid biosynthesis, the epithelial-mesenchymal transition pathway, and a number of cancer signaling pathways. Also, an integrated approach was used to correlate miRNA and mRNA differential profiles of the RNA-Seq data (**S3A Fig**). In hypoxic SLK cells, 426 differentially expressed mRNAs were predicted targets of the 52 modulated miRNAs. The IPA microRNA Filter analysis further revealed that 65% (278 out of 426 mRNAs) of these predicted targets inversely correlated with the changes in miRNA expression (up-regulated miRNA and down-regulated target mRNA, or down-regulated miRNA and up-regulated target mRNA). Thus, substantially more than half of the changes occurring in the mRNA targets under hypoxic stress were consistent with predicted changes in the levels of various miRNAs, suggesting that at least some of these mRNA changes were a result of the changes in miRNA expression. This percentage is similar to that (73%) which was obtained when miRNAs and mRNA targets were similarly analyzed in KSHV-infected SLKK cells as compared with uninfected SLK cells [25]. When we restricted the IPA analysis to targets with a high confidence prediction or experimentally observed responses, 35 miRNAs were identified that were paired to 108 mRNA targets (**S3A Fig**). Specifically, eight miRNAs that were up-regulated by hypoxia were predicted to target 43 mRNAs that were down-regulated, and 27 miRNAs that were down-regulated by hypoxia were predicted to target 65 mRNAs that were up-regulated. These miRNA-mRNA pairs are listed in **S3B Fig**. Notably, changes in miR-210 were correlated with changes in homeobox A1 (HOXA1), a well-described target of miR-210 [37], and CLUH, a regulator of mitochondrial biogenesis [39], among others.

### Relationship between the cellular response to hypoxia and KSHV infection

**Fig 2:**
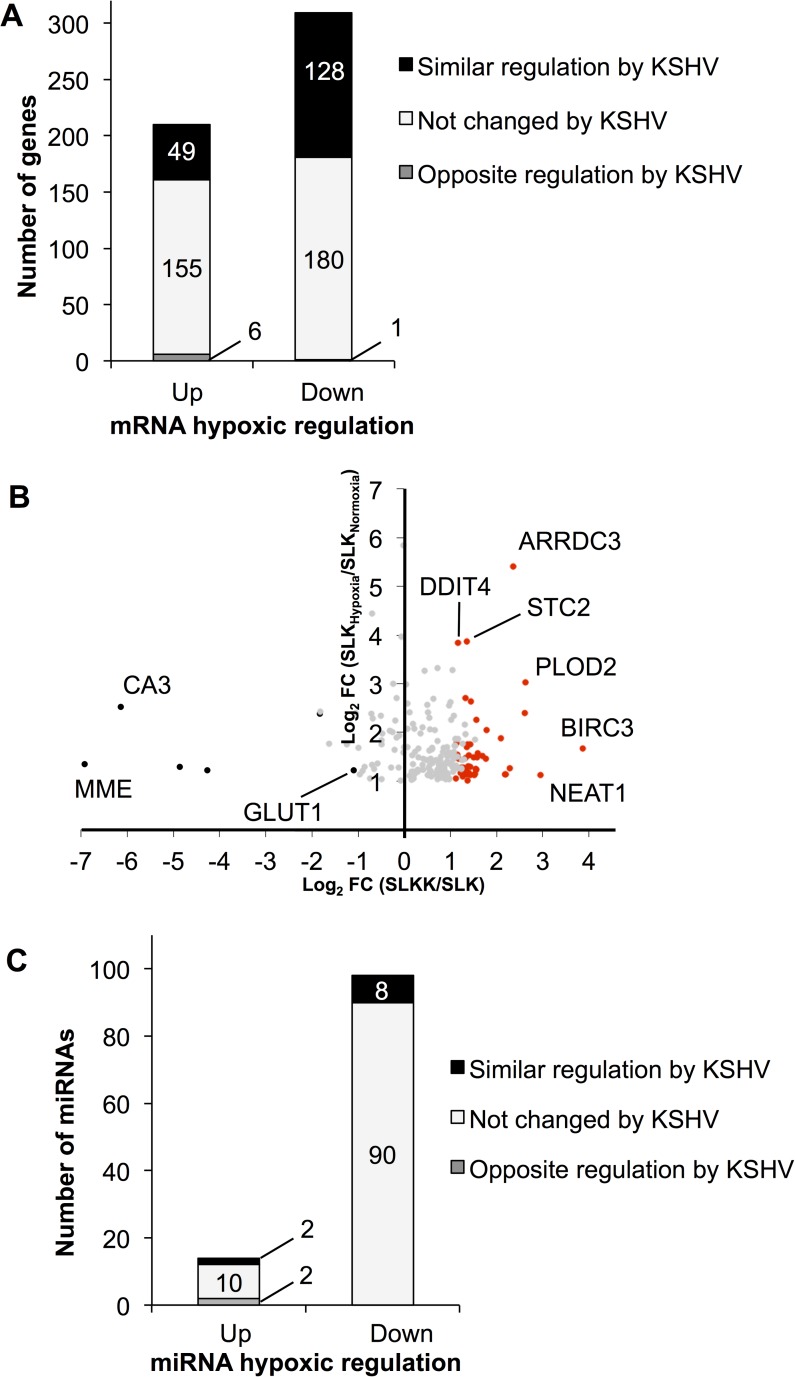
RNA-Seq analysis reveals that the cellular responses to hypoxia and KSHV infection overlap substantially at the mRNA but not at the miRNA level. mRNA and miRNA differential expression in hypoxic vs. normoxic SLK cells was compared with publicly available data from KSHV-infected SLKK vs. uninfected SLK cells [25]. (**A**) Genes that are up-regulated or down-regulated by hypoxia (P≤0.05, FC ≤-2 and ≥2) are similarly regulated (P≤0.05, FC ≤-2 and ≥2) due to KSHV infection. Out of 210 hypoxia-induced genes, ~23% (49) were also up-regulated by KSHV infection (see **S1 Table** for details). Out of 309 hypoxia-repressed genes, ~41% (128) were also down-regulated by KSHV infection [25]. (**B**) Scatter plot showing genes up-regulated by hypoxia and their respective regulation by KSHV infection. This illustrates to what extent the 210 genes that were up-regulated by hypoxia were also changing due to KSHV infection [25]. The x-axis represents the log_2_ fold change (SLKK vs. SLK cells). The y-axis represents the log_2_ fold change (hypoxia vs. normoxia in SLK cells). Red dots depict genes up-regulated by KSHV infection, black dots depict genes that are down-regulated by KSHV infection, and grey dots are those that do not meet criteria for up- or down-regulation. (**C**) miRNAs that are up-regulated or down-regulated by hypoxia (P≤0.05, FC ≤-2 and ≥2) show little overlap with the KSHV infection response. This illustrates to what extent miRNAs that were regulated by hypoxia (*P*≤0.05, FC ≤-2 and ≥2) were also changing with similar cut-off values due to KSHV infection [25]. Out of 14 hypoxia-induced miRNAs, two were also up-regulated by KSHV infection (miR-210 and miR-4671-3p). Out of 98 hypoxia-repressed miRNAs, eight were also down-regulated by KSHV infection (miR-548b-3p, miR-551-3p, miR-579, miR-1270, miR-3136-5p, miR-3180-5p, miR-4793-3p, and miR-5695).

KSHV infection and KSHV-encoded proteins have previously been shown to increase HIF transcription and its responsiveness to hypoxia mimics [16,23,40,41]. With this background, we wanted to explore the relationship between the genes affected by hypoxia and those affected by KSHV infection in SLK cells. To this end, we compared the cellular changes induced by hypoxia in SLK cells to our previous analysis of the differences between KSHV-infected SLKK cells and uninfected SLK cells [25]. SLKK cells are chronically infected by KSHV, and the virus is in a tightly latent state [42]. In **Fig 2,** we compare the effects of hypoxia in SLK cells with those of chronic KSHV infection (SLKK vs. SLK) at both miRNA and mRNA levels. Of the 210 genes that were significantly up-regulated by hypoxia (*P*≤0.05, FC ≤-2 and ≥2), 49 (23%) were also significantly up-regulated by KSHV infection (**Fig 2A**). Also, of the 309 genes that were down-regulated by hypoxia, 128 (41%) were also down-regulated by KSHV infection. Overall, of the 519 hypoxia-regulated genes in SLK cells, 177 (34%) were similarly regulated by KSHV.

The genes up-regulated by both hypoxia and KSHV infection are listed in **S1 Table** and include genes that are known to play roles in hypoxia and viral pathogenesis; i.e. nuclear enriched abundant transcript 1 (NEAT1) [43,44], integrin alpha 5 (ITGAV) [45,46] and baculoviral IAP repeat-containing 3 (BIRC3) [47,48]. While there was considerable overlap, of the 210 genes that were up-regulated by hypoxia (*P* ≤0.05 and FC≥2), a substantial number (155) remained unchanged with KSHV infection and 6 were down-regulated by KSHV (*P* ≤0.05 and FC ≤-2) (**Fig 2A**). To get a better sense of how these hypoxia-responsive genes were affected by KSHV infection, we plotted the KSHV-induced changes in the genes that met our cut-off criteria for up-regulation by hypoxia (*P* ≤0.05 and FC ≥2) (**Fig 2B**). As can be seen, while a number of genes were significantly up-regulated under both conditions (49 red dots in **Fig 2B**), a substantial number of genes up-regulated by hypoxia were also up-regulated with KSHV infection but just not to the level sufficient to meet the cut-off criteria (*P*>0.05) (grey dots to the right of the y-axis in **Fig 2B**). Only 6 genes were up-regulated by hypoxia but significantly down-regulated by KSHV infection (black dots in **Fig 2B**); interestingly, these included the high-affinity glucose transporter GLUT1, a HIF-1α-target gene also known as SLC2A1 that plays a role in glycolysis and the Warburg effect [49]. Consistent with this finding, it has recently been reported that GLUT1 is down-regulated by KSHV in rat mesenchymal cells and that this down-regulation promotes cell survival and oncogenic transformation [50].

Looking at the genes affected by KSHV, a relatively smaller proportion were similarly affected by hypoxia; 177 out of 1,559 (11%) of the genes deregulated by KSHV were similarly changed by hypoxia (**S4 Fig**). Overall, these results suggest that KSHV similarly affects many of the genes affected by hypoxia, although the extent of the change with KSHV infection is often less. In addition, the results demonstrate that while the hypoxic response comprises a substantial portion of the changes in cellular gene expression induced by KSHV, KSHV induces a number of other changes in gene expression unrelated to hypoxia.

A similar comparison was carried out on the data obtained from the small RNA-Seq differential analysis (and Taqman analysis in regard to miR-210). There were only ten deregulated miRNAs that overlapped between hypoxia and KSHV infection (**Fig 2C**). Of these ten miRNAs, two (miR-4671-3p and miR-210) were up-regulated, while the other eight were down-regulated; the most abundant down-regulated miRNAs were miR-548b-3p and miR-1270.

### Similarities between KSHV infection and hypoxia in endothelial cells and AIDS-KS

In order to further explore the relationship between the cellular genes affected by KSHV infection and those affected by hypoxia, we analyzed the reported results of two studies of human umbilical vein endothelial cells (HUVECs) [51,52]. KS spindle cells are thought to be derived from endothelial cells, and HUVECs can thus potentially provide more insight into the pathogenesis of KS than SLK cells. One study used microarray to analyze the genes whose expression was modulated by *de novo* KSHV infection of HUVECs (48 hrs post-infection) [51], while the other used RNA sequencing to analyze HUVECs exposed to hypoxia (1% O_2_) for 48 hrs [52]. Overall, there were 8,863 genes that were analyzed in both studies, of which 350 were dysregulated by *de novo* KSHV infection and 1,137 were dysregulated by hypoxia (using a 1.5 fold cut-off for both parameters). Of the 350 genes that were dysregulated by *de novo* KSHV infection in HUVECs, 49 (14%, including 40 up-regulated and 9 down-regulated) were similarly dysregulated by hypoxia (**S2 Table**). It is worth pointing out that unlike the SLKK cells, in which KSHV infection was almost completely latent, KSHV *de novo* infection of HUVECs involves expression of both latent and certain lytic KSHV genes and in addition involves a cellular response to acute viral infection. Even so, the genes affected by the response to hypoxia comprised a considerable proportion of the genes modulated by *de novo* KSHV infection. Furthermore, we compared the reported expression profile of KS lesions with known hypoxic gene signatures. Cornelissen et al. [53] identified 76 key host genes that are dysregulated in AIDS-KS lesions as compared to normal tissue. Comparing these results to studies of hypoxia, 22 (29%) of these genes are either known HIF-1 targets, have been reported to be similarly dysregulated by hypoxia, or were similarly dysregulated in the SLK/SLKK cell model (**S3 Table**). Of note, one of the genes up-regulated in hypoxia and AIDS-KS, PKM2, has been separately shown to regulate the KS angiogenic phenotype by acting as a coactivator of HIF-1 and increasing the levels of HIF-1 angiogenic factors, including VEGF [54]. Overall, there was a consistent pattern of substantial overlap between KSHV infection and hypoxia.

### Changes in mRNA expression profiles in hypoxic SLKK cells

**Fig 3:**
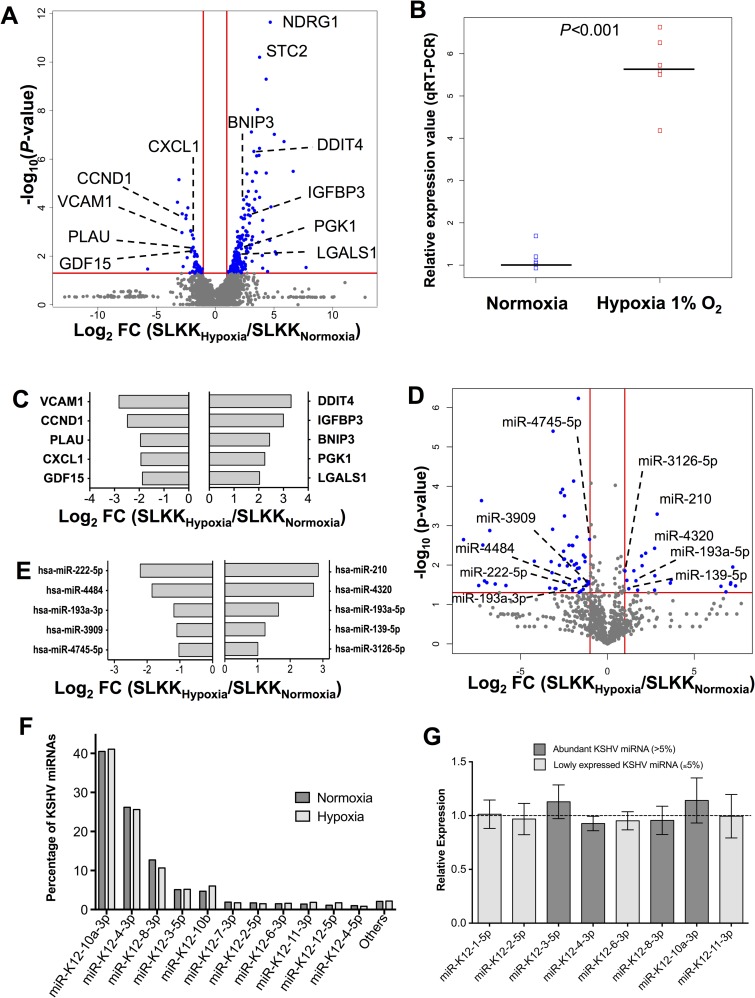
Differential expression of mRNAs and miRNAs in hypoxic vs. normoxic infected SLKK cells. **I**nfected SLKK cells were incubated under standard (normoxic, 21% O_2_) conditions or in hypoxia (1% O_2_) (n = 3). After 24hrs, total RNA was extracted. cDNA libraries for small RNA and mRNA were prepared and sequenced on the Illumina HiSeq platform (Material and Methods). (**A**) Volcano plot of mRNA differential expression in hypoxic vs. normoxic infected SLKK cells. The plot is depicted as in **Fig 1A**, with vertical and horizontal red lines similarly representing the thresholds of a fold change of 2 or -2, and of a *P*-value of 0.05, respectively. Only annotated genes are showed in this volcano plot. Dots in blue depict mRNAs that meet the cut-off values for up-regulation or down-regulation. (**B**) VEGF differential expression in hypoxic vs. normoxic SLKK cells. This plot represents the fold difference in VEGF expression between hypoxia and normoxia in SLKK cells, measured by qRT-PCR assays (n = 6). The plot is depicted as in **Fig 1C**. (**C**) Top 5 most abundant mRNAs up-regulated and down-regulated in hypoxic vs. normoxic infected SLKK cells. Bars left and right of the y-axis show the 5 most abundant mRNAs that are repressed and induced, respectively, in hypoxia compared to normoxia in SLKK cells with *P*-value of ≤0.01 to focus on those with substantial significance. The mRNAs were then ordered by fold change. (**D**) Volcano plot of human miRNA differential expression in hypoxic vs. normoxic infected SLKK cells. The plot is depicted as in **Fig 1A**, with vertical and horizontal red lines similarly representing the thresholds of a fold change of 2 or -2, and of a *P*-value of 0.05, respectively. (**E**) Top 5 most abundant miRNAs up-regulated and down-regulated in hypoxic vs. normoxic infected SLKK cells. Bars left and right of the y-axis show the 5 most abundant miRNAs that are repressed and induced, respectively, in hypoxia compared to normoxia in SLKK cells (*P*≤0.05). The miRNAs were ordered by fold change. (**F**) The expression levels of individual KSHV miRNAs in SLKK cells are shown as the percentage of the total viral miRNA expression. Data are the average of sequenced SLKK samples from three independent experiments. For a detailed breakdown, see **S5 Table**. (**G**) Taqman assays showing relative expression of KSHV miRNAs in hypoxic vs. normoxic SLKK cells. Bars show the relative expression of individual KSHV miRNA in hypoxic SLKK cells as compared to normoxic SLKK cells. The dashed line corresponds to the expression of the normoxic levels. No significant change was observed. Experiments were performed in biological triplicates.

We were further interested in studying how hypoxia affected mRNA and miRNA expression in KSHV-infected cells. To this end, KSHV-infected SLKK cells were exposed to 1% O_2_ for 24hrs, and deep sequencing of mRNA libraries of these hypoxic and control normoxic SLKK cells was performed (each n = 3). Differential analysis using Cufflinks showed that 268 annotated genes were differentially expressed; 215 genes were up-regulated and 53 were down-regulated (*P*≤0.05, FC ≤-2 and ≥2; in blue in **Fig 3A**). In order to confirm that hypoxic induction occurred in the SLKK cells, quantitative real time PCR was again undertaken to determine the expression of VEGF in hypoxia. In the SLKK RNA sequencing, VEGF was up-regulated 3 fold but did not make our significance threshold of *P*≤0.05 (*P* = 0.07). However, as seen in the quantitative real time PCR data in **Fig 3B**, there was a ~5.5 fold induction of VEGF upon exposure to hypoxia (*P*<0.001), confirming that the cells were in fact under hypoxic stress. Of interest, N-Myc downstream regulated 1 (NDRG1) was the most significantly up-regulated gene in hypoxic SLKK cells (**Fig 3A**), with a 4.7 log_2_ fold change increase. These results differed from those found in both normoxic and hypoxic SLK cells, in which NDRG1 was poorly expressed and was thus not significantly changed. NDRG1 expression has been shown to be increased by a variety of environmental stresses, including hypoxia, in either normal or tumor cells, and is involved in caspase activation and apoptosis [55–57]. Also, this is one of the genes that was up-regulated by both hypoxia and *de novo* KSHV infection in HUVECs (**S2 Table**). Another gene of interest in SLK and SLKK cells was stanniocalcin-2 (STC2). This was one of the mRNA most significantly up-regulated by hypoxia in both SLKK cells and SLK cells, with a 3.8 and 3.9 log_2_ fold change increase, respectively (**Figs 1A** and **3A**). STC2 promotes cell proliferation, epithelial-mesenchymal transition (EMT) and invasiveness in hypoxia; traits that likely promote viral persistence and malignant progression [26,58]. After selection based on the highest read count abundance with *P*≤0.01, we determined the 5 most abundant up- and down-regulated mRNAs in hypoxic SLKK cells and then ordered them based on fold change (**Fig 3C**). Of these mRNAs, DDIT4, IGFBP3, BNIP3, PGK1 and LGALS1 were up-regulated, while VCAM1, CCND1, PLAU, CXCL1 and GDF15 were down-regulated. Some of these genes, BNIP3, DDIT4 and IGFBP3 for example, were similarly changing in SLK cells under hypoxic stress.

It was previously shown that KSHV infection leads to enhanced transcription of HIF and increases the induction of HIF-1 and HIF-2 by a hypoxic mimic [23]. Therefore, we wanted to assess the levels of HIFs across all four conditions (i.e. normoxic-uninfected, normoxic-infected, hypoxic-uninfected, and hypoxia-infected samples) in order to determine the degree to which HIF levels might play a role in the observed changes. We were not able to detect HIF-1α in normoxic SLK or SLKK cells, and we could not reliably detect HIF-2α under any conditions. However, consistent with the results of Carroll et al. in endothelial cells infected with KSHV *de novo*, [23], HIF-1α was substantially more up-regulated by hypoxia in SLKK cells than SLK cells (**S5 Fig**), consistent with a relative increase in HIF activity in SLKK cells.

### Hypoxic miRNA response in KSHV-infected SLKK cells

Looking at hypoxia-induced changes in miRNA expression profiles in SLKK cells using the same cutoffs (*P*≤0.05, FC ≤-2 and ≥2), 79 miRNAs were significantly deregulated by hypoxia (**Fig 3D**). Of these, miR-210 was again the most significantly up-regulated miRNA (*P* = 2.0x10^-4^ and log_2_ fold change = 3.7; **Fig 3D and 3E**), similar to uninfected SLK cells. Of these 79 hypoxamiRs (miRNAs regulated by hypoxia), 72 made the abundance cut-off (average miR count ≥1), which allowed for an unbiased, unsupervised clustering of hypoxic vs. normoxic SLKK samples (**S1B Fig**). Of these 72 miRNAs, 51 were down-regulated, while 21 were up-regulated, including miR-210. We selected the 10 most abundant hypoxamiRs (**Fig 3E**). Of these, miR-210, miR-4320, miR-193a-5p, miR-139-5p and miR-3126-5p were up-regulated while miR-222-5p was the most down-regulated miRNA.

We also looked at the effects of hypoxia on KSHV-encoded miRNAs in SLKK cells. Hypoxia was previously shown to affect the expression of LANA mRNA [59], and since at least some KSHV miRNAs share the promoter region with LANA [60], it was possible that their expression might also be affected. However, as seen in **Fig 3F** and **S4 and S5 Tables**, hypoxia did not change the expression of any KSHV miRNAs as assessed by deep sequencing. Also, comparing hypoxic and normoxic SLKK cells, hypoxia did not affect the overall fraction of viral miRNA (~9%) as compared to the total miRNA read count (**S4 Table**). In order to validate these findings, Taqman assays were undertaken to assess the expression of the four most abundant miRNAs in SLKK cells (**Fig 3G**). These included miR-K12-3-5p, miR-K12-4-3p, and miR-K12-8-3p, that are expressed during latency, as well as miR-K12-10a-3p, a KSHV miRNA under the Kaposin promoter that is expressed both during the latent and lytic phases [61]. In addition, we utilized Taqman assays to quantify the expression of four additional KSHV miRNAs that are less abundant in SLKK cells (miR-K12-1-5p, miR-K12-2-5p, miR-K12-6-3p, and miR-K12-11-3p). None of these eight KSHV miRNAs showed a significant change in expression between normoxia and hypoxia in SLKK cells (**Fig 3G**). These results suggest that while certain cellular miRNAs such as miR-210 are dramatically affected by hypoxia and many are down-regulated by hypoxia, none of the KSHV viral miRNAs are affected.

### miR-210 regulation by hypoxia and KSHV

**Fig 4:**
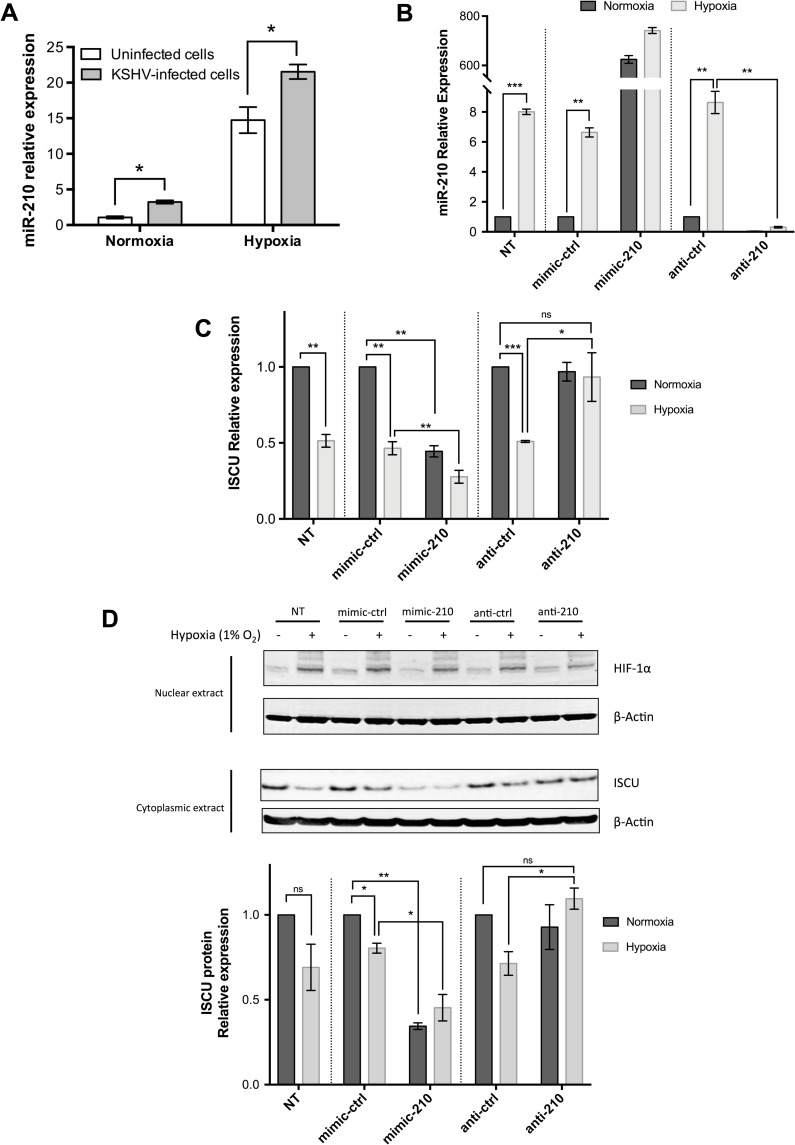
KSHV infection and hypoxia induce miR-210 independently and additively, while miR-210 modulates its target expression, ISCU, in KSHV-infected cells. (**A**) Data represent miR-210 relative expression in normoxic and hypoxic SLKK and SLK cells. Values are normalized to the average of normoxic SLK controls set to one. Taqman assays demonstrate that hypoxic conditions (1% O_2_ for 24hrs) up-regulate miR-210 in both KSHV-infected and uninfected cells (*P* ≤0.01, Student’s t-test). Additionally, Taqman assays show that KSHV infection alone up-regulates miR-210, in both normoxia and hypoxia (*P ≤0.05, Student’s t-test). (**B**) Analysis of miR-210 expression in normoxic and hypoxic SLKK cells transfected with miR-210 mimics, anti-miR-210 inhibitors or controls using Taqman assays. Bars depict the relative expression of miR-210 compared to normoxic NT controls. NT: No Transfection. (**C**) qRT-PCR analysis of ISCU transcript in normoxic and hypoxic SLKK cells transfected with miR-210 mimics, anti-miR-210 inhibitors or controls. Values normalized as in (**B**). NT: No Transfection. (**D**) Quantitative immunoblotting analysis in normoxic and hypoxic SLKK cells transfected with miR-210 mimics, anti-miR-210 inhibitors or controls. (**A**), (**B**), (**C**), and (**D**) depict the mean ± sem of three independent experiments. *, **, and *** indicate *P* ≤0.05, *P* ≤0.01, and *P* ≤0.001, respectively.

Because miR-210 was consistently increased in response to hypoxia and also increased in response to KSHV infection [25], a more detailed investigation of its regulation was undertaken. The relative expression of miR-210 was evaluated by Taqman assay under four different conditions: normoxic SLK cells, normoxic SLKK cells, hypoxic SLK cells and hypoxic SLKK cells (each n = 3). As previously shown by Taqman assay [25], KSHV infection alone increases miR-210 levels (~2.5 fold increase) (**Fig 4A**). In addition, there was a strong induction of miR-210 in hypoxic SLK cells (15–20 fold increase), and levels were highest of all in hypoxic SLKK cells. Based on this observation and miR-210’s role in other cancers, we hypothesize that an increase in miR-210 in KSHV-infected cells, either in normoxia or in hypoxia, may help promote cellular changes that favor infected cells and the development of KSHV-induced cancers.

ISCU, a mitochondrial iron sulfur scaffold protein, is a miR-210 target that is important in cellular metabolism. In MCF7 cells, it has been shown that miR-210-mediated down-regulation of ISCU contributes to a shift to glycolysis, enhanced cell survival, and an increase in iron uptake required for cell growth [62]. To investigate whether ISCU is in fact down-regulated by miR-210 in our cell model, transfection of miR-210 miRNA mimics and miR-210 miRNA inhibitors was performed in SLKK cells, either in normoxia or in hypoxia. These miRNA inhibitors are small single-stranded RNA molecules that bind to and inhibit specific endogenous miRNA molecules in order to down-regulate miRNA activity. As seen in **Fig 4B,** hypoxia significantly increased miR-210 expression by ~6–8 fold in SLKK cells. As expected, the transfection of miR-210 mimics or inhibitors substantially increased or decreased miR-210 relative expression, respectively (**Fig 4B**). Note that after transient transfection with miRNA mimics or antisense inhibitors, the majority of transfected RNA is vesicular and is therefore not accessible for loading into Argonaute [63]. However, the levels of RISC-associated miRNA mimics are close to those of endogenous miRNAs [63]. This data allowed for a further analysis of the effects of miR-210 on expression of its putative target, ISCU. To this end, we evaluated the levels of ISCU by qRT-PCR in SLKK cells under these same conditions (**Fig 4C**). ISCU was found significantly down-regulated after transfection of miR-210 mimics and after exposure to hypoxia (NT control, mimic-ctrl, and anti-ctrl). Finally, ISCU was significantly up-regulated as compared to hypoxic anti-ctrl when miR-210 inhibitors were transfected, as had been described in other cell systems [62]. ISCU protein expression was similar but not completely identical to that of ISCU mRNA (**Fig 4D**). In particular, ISCU protein was significantly reduced when miR-210 mimics were transfected, and significantly increased when miR-210 inhibitors where transfected, demonstrating that it is modulated by miR-210 in SLKK cells. However, ISCU protein expression in hypoxia did not decrease as much as its mRNA counterpart. These results provide evidence that ISCU is a target of miR-210 in KSHV-positive SLKK cells and that miR-210-mediated repression of ISCU could function to mediate cell survival.

### Comparison of the hypoxic response between KSHV infected and uninfected cells

**Fig 5:**
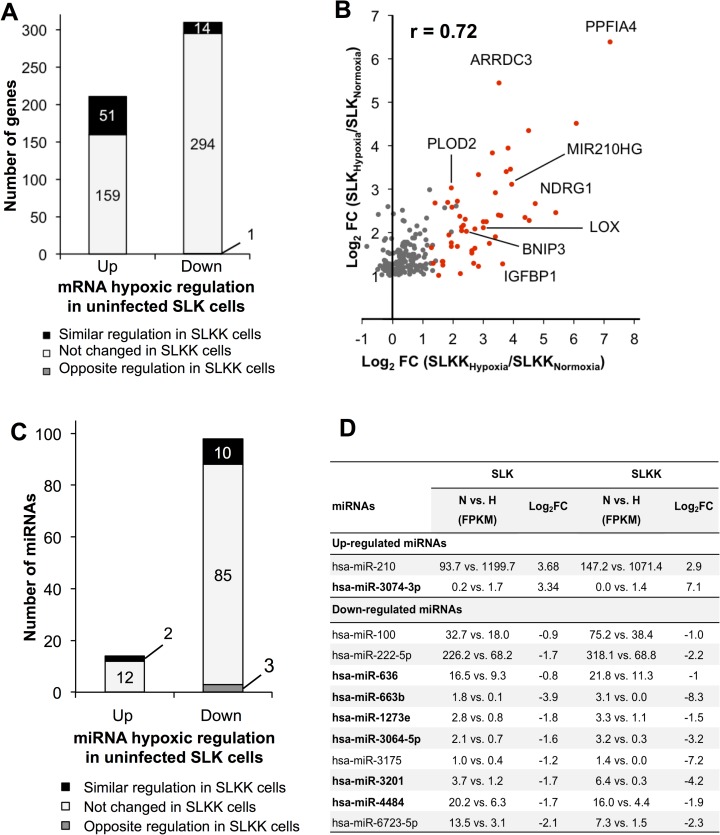
RNA-seq analysis shows the overlap between hypoxia-regulated mRNAs and miRNAs in SLK vs. SLKK cells. Differentially expressed mRNAs and miRNAs in hypoxic vs. normoxic SLK cells were compared to those in hypoxic vs. normoxic SLKK cells. (**A**) This plot illustrates the extent to which genes that were significantly deregulated by hypoxia in SLK cells (P≤0.05, FC ≤-2 and ≥2) were also deregulated by hypoxia in SLKK cells. Out of 210 hypoxia-induced genes in SLK cells, ~24% (51) were also up-regulated in SLKK cells. Out of 309 hypoxia-repressed genes in SLK cells, ~5% (14) were also down-regulated in SLKK cells; and one mRNA, PTGES, was repressed by hypoxia in SLK cells and induced in SLKK cells. (**B**) This scatterplot illustrates the extent to which the 210 genes that were up-regulated by hypoxia in SLK cells were also changed by hypoxia in SLKK cells. The x-axis represents the log_2_ fold change of SLKK_Hypoxia_ vs. SLKK_Normoxia_ cells. The y-axis represents the log_2_ fold change of SLK_Hypoxia_ vs. SLK_Normoxia_ cells. Genes discussed in this manuscript were labelled onto the graph. (**C**) HypoxamiRs in SLK and SLKK cells. The graph illustrates how miRNAs significantly deregulated by hypoxia in SLK cells (*P*≤0.05, FC ≤-2 and ≥2) were changing due to hypoxia in SLKK cells. Out of 14 hypoxia-induced miRNAs in SLK cells, 2 were also up-regulated in SLKK cells (miR-210 and miR-3074-3p). Out of 98 hypoxia-repressed miRNAs in SLK cells, 10 were also down-regulated in SLKK cells; and three miRNAs (miR-135b-3p, miR-33a-3p, miR-4645-3p) were repressed by hypoxia in SLK cells and induced in SLKK cells. See (**D**) for a detailed breakdown. (**D**) This table details the gene expression of miRNAs responding similarly to hypoxia in infected and uninfected cells. miRNA names in bold indicate novel hypoxamiRs. FPKM: fragments per kilobase million. N vs. H: Normoxia versus Hypoxia. Log-transformed fold changes were calculated adding 0.01 to both normoxic and hypoxia FPKM values, in order to correct for nil denominators.

We next compared the cellular response to hypoxia in KSHV-infected versus KSHV-uninfected cells, to explore the effects of latent KSHV infection on the response to hypoxia. When looking at mRNA expression changes due to hypoxia in SLKK and SLK cells, we found that a total of 65 mRNAs, including key hypoxic genes such as BNIP3, LOX, VCAM-1, IGFBP3, PLOD2, and miR-210 host gene (MIR210HG), responded in a similar manner; either induced (51 genes) or repressed (14 genes) by hypoxia in both cell lines (**Fig 5A and 5B**). However, a majority of genes whose expression was significantly modified by hypoxia in SLK cells did not meet the cut-off for change in hypoxia-exposed SLKK cells (**Fig 5A**). To explore this further, we assessed the effects of hypoxia in SLKK cells of genes that were significantly up-regulated by hypoxia in SLK cells (**Fig 5B**). As seen in **Fig 5B** (red dots), a significant portion (24%) of the genes that were significantly up-regulated in SLK cells were also up-regulated in SLKK cells. Most of the other genes (grey dots in **Fig 5B**) that were up-regulated by hypoxia in SLK cells trended towards up-regulation in SLKK cells, but just did not make our cut-off values (*P*≤0.05, FC ≤-2 or ≥2). Interestingly, there were no genes induced by hypoxia in SLK cells but significantly repressed in SLKK cells. This was in contrast to the previous comparison between the effects of hypoxia and KSHV infection in SLK cells, in which some genes up-regulated by hypoxia were significantly down-regulated by KSHV infection (**Fig 2B**). Also, there was a strong positive correlation between the hypoxia-induced mRNA changes in SLK and SLKK cells (Pearson correlation coefficient of r = 0.72). Among the 51 mRNAs up-regulated in both SLK and SLKK cells, BNIP3, a pro-apoptotic gene known for being one of the most hypoxia-responsive genes [64,65], had its mRNA levels increased 4 fold due to hypoxia in both infected and uninfected cell lines. MIR210HG, the host gene for miR-210, was also one of the responders in common between the two cell lines. There were 14 genes that met our cut-off values for down-regulation in both SLK and SLKK cells (**Fig 5A**). VCAM-1 was one of the significantly down-regulated mRNAs in both cell lines, with -1.4 and -2.8 log_2_ FC in SLK and SLKK cells respectively. This is consistent with the fact that hypoxia has been reported to reduce VCAM-1 expression [66,67].

Looking at miRNA expression profiles, only 12 miRNAs were similarly regulated by hypoxia in SLK and SLKK cells (**Fig 5C and 5D**): 2 up-regulated and 10 down-regulated. As expected based on the RNA-Seq studies of SLK and SLKK individually, fewer miRNAs were up-regulated by hypoxia in both SLK and SLKK cells than were down-regulated. The up-regulated miRNAs comprised only miR-210, and miR-3074-3p (**Fig 5D**). Of the 10 down-regulated miRNAs, miR-222-5p had been previously reported down-regulated by hypoxia in MCF7 cells [68]. However, a few down-regulated miRNAs were seen in both SLK and SLKK cells that have not been previously reported down-regulated by hypoxia, including miR-4484 and miR-663b (**Fig 5D**).

### Integrated miRNA-mRNA analysis in hypoxic and normoxic SLKK cells

**Fig 6:**
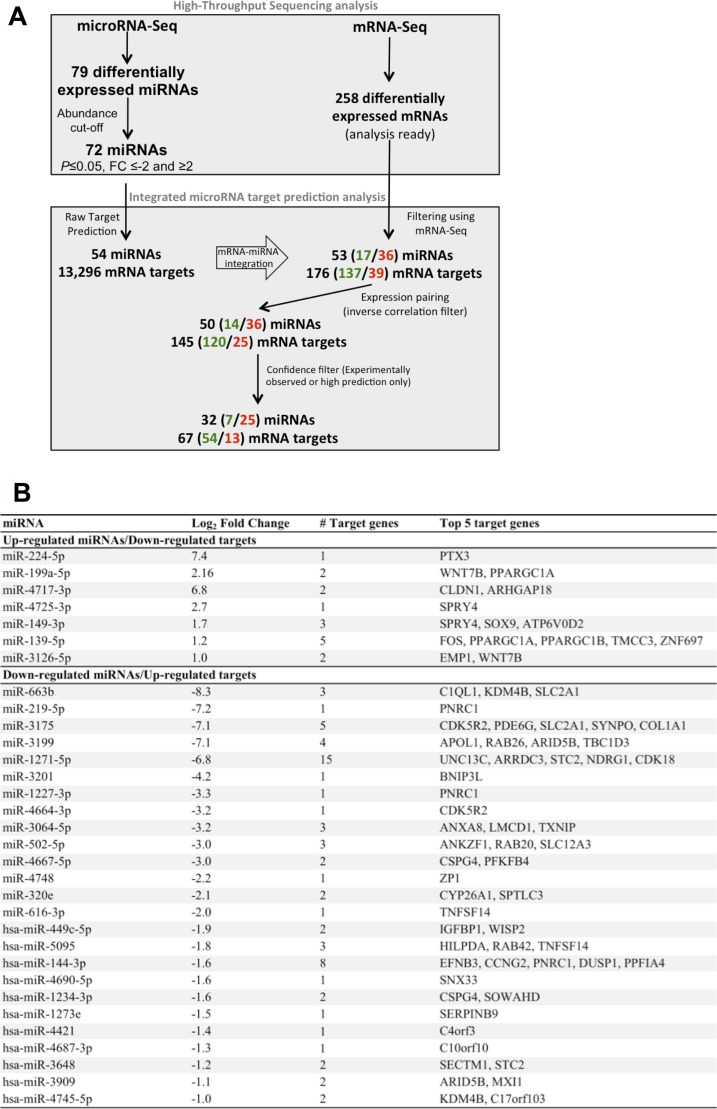
Integration of mRNA-Seq and miRNA-Seq data using Ingenuity Pathway Analysis. (**A**) Workflow of integrated miRNA-mRNA association analysis using IPA. This experimental workflow shows the various filters used to associate miRNA-Seq and mRNA-Seq data from hypoxic and normoxic SLKK cells. Numbers are presented as Total differentially expressed miRNAs or mRNAs (Up-regulated in green/Down-regulated in red). (**B**) Ingenuity analysis predicts inversely correlated miRNA-mRNA target pairs in hypoxic vs. normoxic infected SLKK cells. Using lists of differentially expressed miRNAs and mRNAs as input for the Ingenuity Pathway Analysis (IPA), it was found that 32 differentially expressed miRNAs target 67 mRNAs (high confidence and experimentally observed results). Only mRNAs and miRNAs with opposite differential expression are shown in this table. Columns identify the miRNA name, its log-transformed expression fold change between hypoxic and normoxic SLKK cells, the number of identified targets, and the five most differentially expressed targets.

Ingenuity Pathway Analysis (IPA) was used to identify the key molecular networks that are altered by hypoxia in SLKK cells. The top 15 most significantly altered pathways are illustrated in **S2B Fig**. They include a number of pathways known to be altered by hypoxia such as a number of cancer signaling pathways, glycolysis, Wnt/β-catenin signaling, IL-8 signaling, and retinoic acid receptor (RAR) activation [69–71]. Merging the small RNA-Seq with mRNA-Seq differential analyses, IPA paired 53 miRNAs with 176 mRNA targets, and a remarkable 82% (145 mRNAs) of these targets were concordant under hypoxia (either up-regulated miRNA and down-regulated target, or down-regulated miRNA and up-regulated target; **Fig 6A**), again suggesting that the miRNA changes may be in many cases driving the changes in the target genes. Looking only at the miRNAs and targets that were either paired with a high level of confidence or experimentally observed in the literature (confidence filter), 32 hypoxamiRs remained and were paired with 67 targets (**Fig 6A**). **Fig 6B** lists the seven up-regulated miRNAs and their 13 down-regulated targets, as well as the 25 down-regulated miRNAs and their 54 targets. Of note, miR-224-5p is up-regulated in hypoxia and is predicted to down-regulate pentraxin 3 (PTX3), also known as TNF-inducible gene 14 (log_2_FC = -1.7). PTX3 is known to play a dual role in both protecting cells against pathogens and controlling autoimmunity. Interestingly, three of the down-regulated miRNAs (miR-3064-5p, miR-3175, and miR-3201) and some of their respective up-regulated targets in SLKK cells exposed to hypoxia were similarly deregulated in hypoxic SLK cells (**S3B Fig**).

## Discussion

Viruses, and especially those that cause chronic infections, are attuned to and respond to changes in their target cells. At the same time, viral infection leads to a number of changes in these target cells, some mediated by the virus and others as part of the host response to infection. Previous studies have shown that hypoxia and HIFs can affect KSHV biology and KSHV-induced tumor formation [6,7,59]. Moreover, KSHV infection and LANA, a KSHV-encoded latent gene, have been shown to induce the up-regulation of HIF [16] and at least one HIF-responsive gene (VEGFR1) in newly infected endothelial cells [23]. Also, several HIF-responsive genes related to angiogenesis or metabolism have been shown to be elevated in immortalized HUVECs chronically infected with KSHV [70]. However, these data do not provide a complete picture of the cellular response to KSHV infection and the role that hypoxia plays. To further investigate these relationships, we used RNA-Seq analysis to assess the changes induced by hypoxia in SLK cells and compare them with the changes we had previously found in chronically KSHV-infected SLK cells (the SLKK line) [25]; this paper represents the first global survey of these effects. We found that there was a substantial overlap in the alteration in gene expression induced by KSHV infection and hypoxia. In particular, more than a third (34%) of the genes seen differentially expressed under hypoxia were similarly up- or down-regulated by KSHV latent infection. Also, a majority of the 155 genes that made our cut-off for up-regulation by hypoxia but not for KSHV (**Fig 2A**) demonstrated a trend towards up-regulation in KSHV infection (**Fig 2B**). By contrast, only 5 genes up-regulated by hypoxia showed a down-regulation in KSHV-infected cells. Looking at this from the perspective of KSHV-modulated genes, 11% of these genes are genes modulated in the same way by hypoxia. Overall, these results provide evidence that KSHV commandeers the cell response to hypoxia and that hypoxia-related changes in gene expression comprise a substantial portion of the response to KSHV infection. While levels of HIF-1 were greater in hypoxia-exposed SLKK cells than hypoxia-exposed SLK cells, it was noteworthy that a hypoxic signature was observed in normoxic SLK cells in spite of the fact that we could not detect elevated levels of HIF-1 protein. Further studies would be required to determine to which degree the overlap between the hypoxic response and the KSHV infection is due to KSHV-induced up-regulation of HIF, especially under normoxic conditions.

Cells respond to hypoxia through a number of changes, primarily mediated by HIFs. The systems and pathways that are induced by hypoxia include glycolysis and glucose uptake, growth factor signaling, immortalization, resistance to apoptosis, angiogenesis, and genetic instability [72,73]. It is notable that KSHV has such an effect on hypoxia-related genes, and raises the question as to what evolutionary advantage it provides the virus. Looking at specific genes that are up-regulated by both hypoxia and KSHV infection in our study, a number have activities that can facilitate chronic viral infection or thwart innate anti-viral mechanisms. For instance, baculoviral IAP repeat containing 3 (BIRC3), also known as cellular inhibitor of apoptosis 2 (cIAP-2), is a hypoxia-responsive gene that can confer resistance to apoptosis by interfering with caspases [48]. Here, BIRC3 was induced in SLK cells by both hypoxia (log_2_ fold change of 1.7 or 3.2 fold) and by KSHV infection (14.9 fold change) (**S1 Table**). Interestingly, KSHV protein K15 also up-regulates BIRC3 [47], which suggest that the up-regulation in KSHV infection can occur through both HIF-dependent and independent mechanisms. By interfering with apoptosis, BIRC3 can inhibit the ability of KSHV-infected cells to destroy themselves (and the infecting virus) [74–76]. Another gene that is up-regulated by both hypoxia and KSHV infection is NEAT1, a host long non-coding RNA involved in nuclear paraspeckle formation. There is evidence that NEAT1 plays an important role in regulating gene expression [77,78]. NEAT1 has also been found to increase the survival of cancer cells [43], and may thus benefit KSHV infection by preventing the death of KSHV-infected cells.

Up-regulation of genes such as BIRC3 or NEAT1 would presumably be beneficial for most viruses, and in fact several other chronic viruses, including hepatitis B virus, human papillomavirus, Epstein Barr virus, and human T cell lymphotropic virus (HTLV-1) have also been reported to activate HIF and certain HIF-responsive genes. At the same time, the modulation of HIF-responsive genes by KSHV is particularly robust. Endothelial cells are important target cells for KSHV infection, and it is possible that the virus evolved to up-regulate hypoxia-responsive genes in part because several of these genes, including STC2 and IGFBP3 (see **S1 Table**), promote angiogenesis and the growth of endothelial cells [26,79,80]. An unintended consequence of the activation of these and other HIF-responsive genes may be tumorigenesis leading to the development of Kaposi sarcoma and other KSHV-induced tumors.

As described in our previous paper [25], we used SLK and SLKK cells in this study because of the extremely tight control of KSHV latency in these cells and because they provide the ability to compare the effects of hypoxia and KSHV infection on KSHV infected and uninfected cells. SLK cells were originally thought to be derived from a KS biopsy and have a number of endothelial-like features [81]; however, this line was subsequently found to be indistinguishable from Caki-1, a clear-cell renal carcinoma line [81]. Nonetheless, this line remains a valuable tool to study the effects of latent KSHV infection. To further explore whether there was substantial overlap in the cellular response to KSHV infection and to hypoxia in systems that were biologically more similar to KS, we used published datasets to analyze gene changes in HUVECs and in KS lesions, and found evidence in these systems as well. In the *de novo* infected HUVECs system, it is noteworthy that a strong hypoxic signature was seen in the cellular response in spite of the fact that lytic KSHV genes and an acute cellular response to viral infection are present. However, there may be limitations to that system in that HUVECs were infected *de novo* for 48hrs, which corresponds to an early lytic activation. In that phase, KSHV is establishing initial infection and cells are reacting to this infection [82]. Therefore, it is not surprising that cellular genes expressed during this short-lived and transient phase might be different from those that will be differentially expressed during latency and that hypoxia-related genes comprise a smaller percentage of the genes that are differentially expressed. Additionally, compiled results from a number of studies show that a substantial proportion of genes whose expression is modulated in KS lesions are genes that are responsive to hypoxia. Taken together, the prominence of a hypoxic gene activation in KSHV infection and the potential of HIFs to activate KSHV genes may provide a rationale for targeting hypoxic pathways as a potential therapeutic strategy for KSHV-related diseases. Indeed, HIF-1 dysregulation is known to fuel both angiogenesis and tumor metabolism in KS [54]. Furthermore, HIF-1 suppression has been found to lower viral interleukin-6 levels, lytic replication of KSHV, and proliferation of PELs [83], supporting that HIF-1 is indeed an important factor in the maintenance of KSHV infection and the survival of PELs.

A somewhat different picture emerged when we examined the miRNA changes induced by KSHV infection and hypoxia. The majority of the deregulated miRNAs in either hypoxia or in KSHV infection were repressed (88% by hypoxia and 73% by KSHV infection) [25]. However, there was relatively little overlap in the miRNA expression profiles affected by hypoxia and those affected by KSHV infection. A notable exception to this was miR-210, which was significantly up-regulated by both hypoxia and KSHV latent infection; this was confirmed using independent Taqman validation assays. miR-210 is directly up-regulated by the binding of HIF-1α to an HRE in its promoter region and has previously been shown to be the main miRNA induced by hypoxia [33,35] [84]. While miR-210’s effects on KSHV were not investigated here, miR-210 has been extensively studied and is known to be an important factor in regulating the immune response, glycolysis, and tumorigenesis, all areas which may impact the biology of the virus [85,86]. Additionally, miR-210 has been shown to be up-regulated by EBV infection [87] and HIV infection [88], and miR-210 helps maintain virion production in HBV infection [89]. An important target of miR-210 is ISCU, a mitochondrial iron sulfur scaffold protein involved in cellular metabolism [62], and as seen here, changes in ISCU are consistent with its regulation by miR-210 in KSHV-infected cells (**Fig 4C**). Of note, inhibition of ISCU by miR-210 in MCF7 cells has previously been shown to lead to a shift to increased glycolysis, an enhanced cell survival rate, and an increased iron uptake required for cell growth [62]. Such changes could help promote the Warburg effect that has been described in KSHV-infected cells and favors their cell growth and survival. The exact role of miR-210 in KSHV infection and disease pathogenesis remain to be established in future studies.

By contrast to miR-210, the majority of miRNAs affected by hypoxia or by latent KSHV infection were down-regulated. The mechanism leading to these changes is not clear, but could be related to changes in post-translational miRNA biogenesis. In this regard, it has been reported that the key proteins of the miRNA biogenesis, Dicer and Drosha, can be down-regulated in hypoxia, hence reducing miRNA processing [90]. However in the present experiments, neither Dicer nor Drosha mRNAs were found significantly down-regulated by hypoxia in the RNA-Seq data; nevertheless, the levels of these miRNA processing proteins might still be affected by post-transcriptional alterations. Additional studies will be needed to understand this general miRNA down-regulation by hypoxia.

Certain cells newly infected by KSHV, such as endothelial cells, have been reported to increase glycolysis, also known as the Warburg effect, and a number of genes involved in glycolysis are up-regulated by HIF-1 (but not HIF-2) [32,91]. While the increase in miR-210 levels could contribute to the Warburg effect, it was noteworthy that a number of genes involved in glycolysis were up-regulated by hypoxia (including GLUT1, hexokinase 2, phosphoglycerate kinase, pyruvate dehydrogenase kinase-1, and lactate dehydrogenase-5), but were not by KSHV infection. In fact, GLUT1, which transports glucose into the cell, was one of the 5 down-regulated genes in SLKK cells (**Fig 2B**). Interestingly, while GLUT1 is up-regulated in endothelial cells acutely infected by KSHV [54], it has recently been reported to be down-regulated in acutely infected rat mesenchymal cells [50]. It should be noted that SLKK cells are chronically infected and that KSHV is tightly latent in these cells; additional studies will be needed to clarify the effects of KSHV infection on genes involved in glycolysis.

KS preferentially arises in the feet, which have relatively low oxygen tension, and PEL usually arises in pleural spaces, which have no blood vessels and are also hypoxic [9,10]. Previous studies by our group and others have shown that hypoxia and HIFs can activate a number of KSHV genes, including RTA, LANA, and ORFS 34–37. Thus, the combination of KSHV infection and hypoxia appears to be important in the pathogenesis of KSHV-associated tumors. To explore these relationships further, we analyzed the effects of hypoxia on KSHV-infected cells. There was substantial overlap in the affected mRNAs in SLK and SLKK cells exposed to hypoxia, especially considering the genes that were up-regulated. However, in many cases, genes that were moderately up-regulated by hypoxia in SLK cells fell below our significance threshold for up-regulation in hypoxia-exposed SLKK cells (**Fig 5B**), and none of the genes that were up-regulated by hypoxia in SLK cells were down-regulated by hypoxia in SLKK cells (**Fig 5A**). In our RNA-Seq analysis. we did not directly compare the overall changes in mRNA and miRNA in uninfected SLK cells in normoxia to SLKK cells in hypoxia. Such a comparison was made for miR-210 (**Fig 4A**) and revealed that the effects were cumulative. Also, this analysis showed that the induction of miR-210 by hypoxia in SLKK cells was less than that in SLK cells, largely because of the baseline elevation in the SLKK cells. This suggests that the reason that less genes overall were deregulated by hypoxia in SLKK cells than in SLK cells (519 vs. 268) may be because of a similar shift in baseline gene levels by KSHV.

KSHV encodes its own miRNAs, which are formed from 12 precursor miRNAs. These precursor miRNAs are on mRNA transcripts downstream of LANA, and share promoter regions with Kaposin. LANA, Kaposin, and most of the KSHV miRNAs are produced during latency, and some of the miRNAs (e.g. miR-K12-10 and -12) are up-regulated during chemical induction of KSHV lytic infection [92]. Since LANA and the lytic switch gene RTA can be up-regulated by hypoxia and HIF [59], we thought it plausible that expression of at least some of the KSHV-encoded miRNAs might be similarly induced by exposure to a low-oxygen environment. However, the levels of these mature miRNAs were remarkably unchanged by hypoxia (**Fig 3F and 3G**). Some of the KSHV miRNAs are extremely abundant and the combination of a longer half-life of miRNAs compared to the mRNAs [93] might explain this observation. Another factor might be the length of hypoxia, which might be much longer in the context of KSHV infection in humans, compared to the experimental hypoxia treatment in our assays (24hrs). We did not specifically analyze the levels of the miRNA precursors, and it is possible that an increase in these precursors was counterbalanced by a suppression of miRNA maturation, which also contributed to the overall decrease in cellular miRNAs (**Fig 3D**). Additional studies will be needed to clarify this; however, the results indicate that a modulation of KSHV-encoded miRNAs does not have a substantial effect on the response of KSHV-infected cells to hypoxia.

In summary, this global gene survey demonstrates that there is a substantial overlap between the genes affected by hypoxia and by KSHV infection, and shows that induction of a hypoxic gene response is a substantial component of the modulation of cellular genes by KSHV infection. However, by contrast to mRNA expression, there was relatively little overlap in the modulation of miRNAs by KSHV infection and hypoxia, a notable exception being an up-regulation of HIF target miR-210, which has been previously shown to be important in the angiogenic response and in tumor formation. This study provides a more thorough understanding of the role of hypoxia and the interplay between KSHV infection and HIF/hypoxia, and lays the groundwork for further analyses of these interactions.

## Materials and Methods

### Cell culture

Human KS-derived SLK and SLKK cells (also known as SLK+rKSHV.219) [51,81,94,95] were a gift from Dr. Don Ganem (UCSF, CA). They were expanded on receipt, frozen in liquid nitrogen, and stored in a cryogenic tank until used in the experiments described hereafter. Cells were thawed and maintained in Dulbecco’s Modified Eagle medium supplemented with 10% v/v fetal bovine serum (Sigma-Aldrich, St Louis, MO) and 1% Penicillin/streptomycin/glutamine solution (Gibco, Carlsbad, CA). Additionally, KSHV-positive SLKK cells were periodically grown under selection with 10 μg/mL puromycin to maintain the viral episome. Not counting the time during which cells were frozen, SLK cells had been kept in culture for less than 3 months before being used for RNA isolation (RNA-Seq), transfection assays, or *de novo* KSHV infections. SLK cells infected with recombinant rKSHV.219 (SLKK cells) were originally selected over time using puromycin in order to reach a high latent viral expression. Therefore, again not counting the time that they were frozen, the SLKK cells were kept in culture for a longer period of time (6 months to a year) before being used for RNA isolation (RNA-Seq) and transfection assays. All experiments were done in triplicate independent cell cultures maintained at 37°C in humidified 5% CO_2_.

### Hypoxic treatment

Exposure of cell cultures to 1% oxygen was undertaken in an InVivo_2_ Hypoxia Work Station (Ruskinn Technology, UK). This was undertaken in parallel with cells maintained in normoxic conditions (21% O_2_). All cells were harvested for protein and RNA 24hrs post-treatment. All experiments were done at least in triplicate from independent cell cultures.

### Transfection of miR-210 mimics and anti-miR-210 inhibitors

SLK and SLKK cells were seeded at a concentration of 6 to 9x10^5^ cells in a10cm dish in DMEM medium with 10% FBS containing no antibiotics; this allowed cells to achieve 50% confluence the following day. The culture medium was then removed and the cells were transfected with the miRNA mimics (i.e. miR-210), miRNA inhibitors (i.e. anti-210) or negative controls (Dharmacon, Lafayette, CO, USA) at 10nM final concentration, using Dharmafect 1 (Dharmacon). Dharmafect 1 is a potent transfection reagent that allows the negatively charged membrane to interact with the liposome/nucleic acid complex. The miRNA transfection experiments were performed in a 2.4mL Opti-MEM/Dharmafect mixture and 9.6mL DMEM medium (Invitrogen). The cells were incubated in normoxia or hypoxia (1% O_2_) for 48hrs at 37°C/5% CO_2_ before performing RNA isolation and nuclear/cytoplasmic extraction.

### RNA isolation

Total RNA was extracted from cells using miRVana miRNA isolation kit according to manufacturer’s instructions (Ambion, Life Technologies, Carlsbad, CA). RNA abundance and integrity were determined after isolation using a Nanodrop-ND-1000 spectrophotometer (Thermo Fisher Scientific, Waltham, Massachusetts, USA) and an Agilent 2100 Bioanalyzer (Agilent Technologies, Santa Clara, CA), respectively. Only samples of total RNA with an RNA integrity number (RIN) >9 were further used for RNA-sequencing and small RNA-sequencing. All samples were stored at -80°C.

### Quantitative real-time PCR assays

For miRNA expression, stem-loop qPCR was performed using TaqMan Universal master mix (Applied Biosystems) and the following microRNA assays: 000512 for miR-210-3p, 197204 for miR-K12-1-5p, 197192 for miR-K12-2-5p, 008316 for miR-K12-3-5p, 197240 for miR-K12-4-3p, 008459 for miR-K12-6-3p, 241994 for miR-K12-8-3p, 008504 for miR-K12-10a-3p, and 008562 for miR-K12-11-3p. Reference RNA RNU43 (assay 001095) was used as endogenous control to normalize expression. Thermal cycling conditions included an enzyme activation step (95°C for 10 min) and 40 cycles of amplification at 95°C for 15s followed by 60°C for 1min.

For gene expression, cDNA synthesis was performed on total RNA using the Superscript II reverse transcriptase kit, 0.5mM dNTP set and 50 ng/μL random hexamer (Invitrogen). qPCR was performed using the FastStart Universal SYBR Green/ROX master mix (Roche Applied Science, Mannheim, Germany). 18S transcript was used as endogenous control to normalize expression. Thermal conditions included an enzyme activation step (95°C for 10min), 40 cycles of amplification at 95°C for 15 sec and 60°C for 1 min, and melting curve analysis following instrument standard instructions. The following primer sets were used: 5’-CCTTGCTGCTCTACCTCCAC -3’ and 5’- AGCTGCGCTGATAGACATCC-3’ for VEGF, and 5’-GCCCGAAGCGTTTACTTTGA-3’ and 5’-TCCATTATTCCTAGCTGCGGTATC-3’for 18S. All qPCR reactions were performed on an ABI 7300 Real Time PCR instrument (Applied Biosystems). Each experiment was performed in triplicate. Change in miRNA or mRNA expression was determined based on the delta Ct method [96]. Error bars in relative expression plots represent the standard error of the mean and *P*-values were calculated using two-tailed Student t-test unless otherwise stated. *, **, and *** indicate *P* ≤0.05, *P* ≤0.01, and *P* ≤0.001, respectively.

### Protein extraction and western blot analysis

Cells were lysed and cytoplasmic and nuclear extracts were prepared using the NE-PER extraction kit with 1X halt-protease inhibitors cocktail (both Pierce, Waltham, MA) and EDTA. Protein extracts were stored at -80°C. 20μg of protein were mixed with warm 2X lithium dodecyl sulfate (LDS) such that the LDS to sample ratio was 1:4 (v/v). Sample were denatured for 5 minutes at 95°C and subjected to SDS-PAGE (4–12% NuPAGE Bis-Tris) with 1X MOPS running buffer diluted with dH_2_O (all Invitrogen). The proteins were transferred onto a nitrocellulose membrane by an iBlot apparatus for 8 minutes (all Invitrogen). The membrane was blocked with 5% w/v nonfat dry milk in 1X TBST (10nM Tris-HCl, pH 8.0, 150nM NaCl, and 0.05% Tween 20). The blot was incubated with mouse antibodies to HIF-1α (1:500, BD Biosciences, San Jose, CA) or ISCU (1:800, ab180532, Abcam) overnight and goat anti-mouse IRDye 800CW antibody (Licor Biosciences, Lincoln, NE) at a 1:10,000 dilution for 30 minutes; being washed twice with washing buffer in between incubations. Scanning was performed using the Li-Cor Odyssey CLx imaging system coupled with the Image Studio software. Images were later adjusted for contrast and intensity using PowerPoint 2011 version 14.6.3 for Macintosh (Microsoft Co, Redmond, WA).

### Preparation of microRNA libraries for deep sequencing

Small RNA libraries were constructed as previously described [68] using the TruSeq RNA Sample Prep kit (Illumina inc., San Diego, CA). Briefly, after running 2 μg of total RNA in a 15% urea-TBE gel (Invitrogen, Life technologies, Carlsbad, CA) for 1 hr at 200V, the 20 to 30 nucleotide RNA fraction was excised and eluted in 0.3 M NaCl. Following separation of the elute from the gel debris using a Spin-X-column (Thermo Fisher Scientific), the small RNA samples were precipitated in 100% ethanol and 1mg/mL glycogen, incubated at -80°C for 30 min, centrifuged at 14,000rpm for 25 min, washed with 75% ethanol, air dried and resuspended in RNAse-free water. Illumina TruSeq libraries were then prepared according to the manufacturer’s protocol and the final RNA library concentration was measured by a Qubit 2.0 fluorometer using Qubit dcDNA HS Assay kit (Life Technologies). We verified the size of the products contained in the libraries using a high sensitivity DNA chip and an Agilent 2100 Bioanalyzer (Agilent Technologies). Finally, a total of six miRNA libraries (three SLK and three SLKK samples) were sequenced using the Illumina HiSeq platform.

### Preparation of polyadenylated mRNA libraries for deep sequencing

Total RNA of samples used for small RNA-Sequencing was treated with Turbo DNA-free DNase I and Dynabeads (both from Ambion, Life Technology) to deplete samples from the residual DNA and to isolate the polyadenylated mRNA transcriptome, respectively. PolyA+ RNA libraries were then prepared with the ScriptSeq v2 RNA-Seq kit (Epicentre, Madison, WI). The final concentration and size distribution of the RNA libraries were measured by using a Nanodrop-ND-1000 spectrophotometer (Thermo Fisher Scientific), and by running a DNA 100 chip on an Agilent 2100 Bioanalyzer (Agilent Technologies). Finally, a total of six polyadenylated mRNA libraries (three SLK and three SLKK samples) were sequenced using the Illumina HiSeq platform.

### Bioinformatic analysis

For small RNA sequencing, reads were aligned against known miRNAs from miRbase (version 19.0) using the software package SHRIMP [97] (http://compbio.cs.toronto.edu/shrimp/README) with miRNA specific settings (-o 1 -n 2 -r 30% -h 50%—local -Q—qv-offset 32—sam). To process paired-end sequencing, reads were aligned separately covering the mature miRNA both on the forward and reverse read and the obtained number of matches were averaged. The match counts were normalized and tested for differential expression using the edgeR package [98] with default settings. For mRNA sequencing, adapter sequences were trimmed using the Fastx toolkit. Reads were aligned against two target genomes, human (hg19) and KSHV (Genbank accession number NC_009333.1) using TopHat [99] to generate spliced alignments. Transcripts were assembled using Cufflinks and Cuffdiff [100] in order to reveal differentially expressed genes. To be more conservative and eliminate infinite fold differences in cases where there was a nil denominator, fold changes were calculated adding 0.01 read to both the numerator and denominator. Significant mRNA fold change was determined by an adjusted P-value lower than 0.05 based on the Benjamini and Hochberg multiple testing correction. In order to visualize miRNA-Seq profiles, sequencing reads were converted to bedGraph format using BEDtools [101]. The output files were then uploaded and displayed using the UCSC Genome Browser (http://www.genome.ucsc.edu). R (http://www.R-project.org) suite software was used for statistical analyses, heat maps and scatter plots.

### Pathway analysis and inverse correlation study of miRNA and mRNA expression levels

Pathway analysis and inverse correlations between expression levels of differentially expressed miRNAs and their respective target mRNAs were analyzed using Ingenuity Pathway Analysis (IPA, Ingenuity Systems, Redwood City, CA; www.ingenuity.com). This *in silico* analysis software reveals enrichment for molecular networks and signaling pathways. Moreover, we generated medium to high confidence miRNA target predictions and as well as experimentally observed miRNA-mRNA interactions using the IPA tool called “MicroRNA Target Filter” that integrates multiple target prediction algorithms such as TargetScan, TarBase, miRecords and the Ingenuity Knowledge Base. This allowed for the integration of miRNAs with mRNA targets, predicted and validated, that were both differentially expressed in SLKK vs. SLK cells. Opposite expression pairing between miRNA and mRNA levels was implemented to further refine the analysis. Further filtering options were applied such as the confidence parameter.

### Dataset analysis and comparison

We compared two publicly available datasets from published HUVECs studies [51,52]. One study used microarray to analyze the genes modulated by *de novo* KSHV infection (48 hrs post-infection) [51], while the other used RNA sequencing to analyze HUVECs exposed to hypoxia (1% O_2_) for 48 hrs [52]. We parsed the data based on expression fold change (FC>1.5), and an additional *P*-value cut-off of 5x10^-5^ was used on the RNA-seq study [52] Overall, there were 8,863 genes that were detected in both studies, of which 350 and 1,137 were dysregulated by *de novo* KSHV infection and hypoxia, respectively. Additionally, we compared 76 genes known to be dysregulated in AIDS-KS lesions [53] with our results in the SLK/SLKK model and other published hypoxia studies [32,102–117]. There were 22 out 76 genes similarly regulated in AIDS-KS and hypoxia.

### Data availability

Raw miRNA and mRNA data are available on the NCBI Gene Expression Omnibus (GEO) database under the series accession identifier GSE79032.

## Supporting Information

S1 Fig Expression of hypoxia-regulated miRNAs (hypoxamiRs) in the SLK/SLKK model. (**A**) Expression of hypoxamiRs in uninfected SLK cells. Heat map of miRNA changes between hypoxic and normoxic SLK cells in the six individual replicates (three normoxic and three hypoxic SLK samples). Presented is the relative expression of 47 significantly deregulated cellular miRNAs in hypoxic vs. normoxic infected cells (*P*-value ≤0.05, linear FC ≤-2 or ≥2) with an average read count ≥1. Using an uncentered Pearson correlation as the distance metric, an unsupervised hierarchical heatmap was generated, with each row represents a miRNA and each column representing a biological replicate. Red, black and green denote low, median and high relative miRNA expression, respectively. (**B**) Expression of hypoxamiRs in infected SLKK cells. Heat map of miRNA changes between hypoxic and normoxic SLKK cells in the six individual replicates (three normoxic and three hypoxic SLKK samples). Presented is the relative expression of 72 significantly deregulated cellular miRNAs in hypoxic vs. normoxic infected cells (*P*-value ≤0.05, linear FC ≤-2 or ≥2) with an average read count ≥1. The plot is depicted as in **S1A Fig**, with each row representing a miRNA and each column representing a biological replicate. Red, black and green denote low, median and high relative miRNA expression, respectively.
(PDF)

S2 Fig Pathway analysis of hypoxic SLK and SLKK cells. (**A**) Top 15 pathways altered by hypoxia in SLK cells. A list of differentially expressed mRNAs was used as input for Ingenuity Pathway Analysis, generating pathways altered by hypoxia in uninfected SLK cells. The 15 pathways that are most significantly altered are illustrated here. The yellow line reflects the significance (*P* ≤0.05 when–log(*P*-value) >1.3). The percentage of down-regulated and up-regulated genes in a given pathway is depicted in green and red, respectively. (**B**) Top 15 pathways altered by hypoxia in SLKK cells. A list of differentially expressed mRNAs in SLKK cells was used for Ingenuity Pathway Analysis. The 15 pathways that were most significantly altered by hypoxia are presented here. For any given pathway, the percentage of down-regulated and up-regulated genes are depicted in green and red, respectively. The total number of genes in each pathway is indicated in bold on the right hand side. An asterisk * indicates pathways that were also found altered due to infection alone [25]. The symbol (¶) indicates pathways that were also found altered by hypoxia in uninfected SLK cells. The significance of each pathways altered by hypoxia in SLKK cells was assessed by *P*-value.
(PDF)

S3 Fig Ingenuity Pathway Analysis using mRNAs and miRNAs of hypoxic vs. normoxic uninfected SLK cells. (**A**) Workflow of integrated miRNA-mRNA association analysis using IPA. This experimental workflow shows the various filters used to associate miRNA-Seq and mRNA-Seq data from hypoxic and normoxic SLK cells. Numbers are presented as Total differentially expressed miRNAs or mRNAs (Up-regulated/Down-regulated). (**B**) Ingenuity analysis predicts inversely correlated miRNA-mRNA target pairs in hypoxic vs. normoxic uninfected SLK cells. Using lists of differentially expressed miRNAs and mRNAs as input for the Ingenuity Pathway Analysis (IPA), it was found that 35 differentially expressed miRNAs target 108 mRNAs (high confidence and experimentally observed results). Only mRNAs and miRNAs with inverse differential expression are shown in this table. Columns identify the miRNA name, its log-transformed expression fold change between hypoxic and normoxic SLK cells, the number of identified targets, and the five most differentially expressed targets. In bold are miRNAs and mRNA targets that have been previously reported in the literature.
(PDF)

S4 Fig Genes and miRNAs regulated by KSHV. This illustrates to what extent genes (**S4A**) and miRNAs (**S4B**) that are regulated by KSHV infection [25] are also changing due to hypoxia. The analysis is done as in **Fig 2A**.
(PDF)

S5 Fig HIF-1α regulation in hypoxic and normoxic SLKK and SLK cells. We determined the level of HIF-1α protein in the cytoplasmic and nuclear fractions of SLK and SLKK cells, under hypoxia and normoxia. C and N stand for cytoplasmic and nuclear fraction, respectively. **S5A** shows Western Blot analyses of HIF-1α, while **S5B** shows the intensities of immunoreactive bands quantified by densitometric analysis.
(PDF)

S1 Table Genes up-regulated in hypoxia and KSHV infection in the SLK/SLKK model. These 49 genes are up-regulated by both hypoxia and KSHV infection. The 5 most abundant mRNAs (average read count) appear in bold (DDIT4, IGFBP3, CLIC4, RSPS27L and NEAT1).
(PDF)

S2 Table Genes up-regulated in hypoxic HUVECs and KSHV *de novo* infected HUVECs. These 40 genes are up-regulated by both hypoxia and KSHV *de novo* infection (48 hrs post infection) in HUVECs [51,52]. NDRG1, which was also found up-regulated in the SLK/SLKK cell model, is in bold.
(PDF)

S3 Table Genes similarly dysregulated in AIDS-KS lesions and in hypoxia. These 22 genes are similarly regulated in AIDS-KS and in hypoxia. Hypoxic signatures from SLK cells, SLKK cells, HUVECs [52], as well as other cell lines [32,102–117] were compared to the 76 key host genes dysregulated in AIDS-KS [53]. Also, HMOX1, DUSP1 and LGALS1 were significantly induced by hypoxia in SLKK cells, and TXNIP was up-regulated in both SLK and SLKK hypoxic cells.
(PNG)

S4 Table Read statistics for human and viral miRNAs expressed in hypoxic and normoxic SLKK cells. Three independent experiments are displayed (A, B, and C). Columns identify the replicate number, the number of aligned miRNA reads per species and total, and the percentage of KSHV miRNA reads vs. the total number of aligned reads for each SLKK replicate and overall.
(PDF)

S5 Table Detailed analysis of KSHV miRNA read counts in hypoxic and normoxic SLKK cells. The average of three independent experiments for each condition is displayed. Columns identify each KSHV miRNA, its total miR count, its percentage as compared to either KSHV miR reads or the overall read number, in either normoxia or hypoxia. The top part illustrates KSHV miRNAs present in more than 1% of total KSHV reads. The rest is displayed under “Others”. KSHV miRNAs in bold have been validated by Taqman assays (see **Fig 3G**).
(PDF)
