# Supplementary figures and images for: RNA Sequencing Reveals that Kaposi Sarcoma-Associated Herpesvirus Infection Mimics Hypoxia Gene Expression Signature
Source: PLoS Pathog. 2017 Jan 3;13(1):e1006143. doi: 10.1371/journal.ppat.1006143 (PMC5234848; doi:10.1371/journal.ppat.1006143)

## S1 Figure.

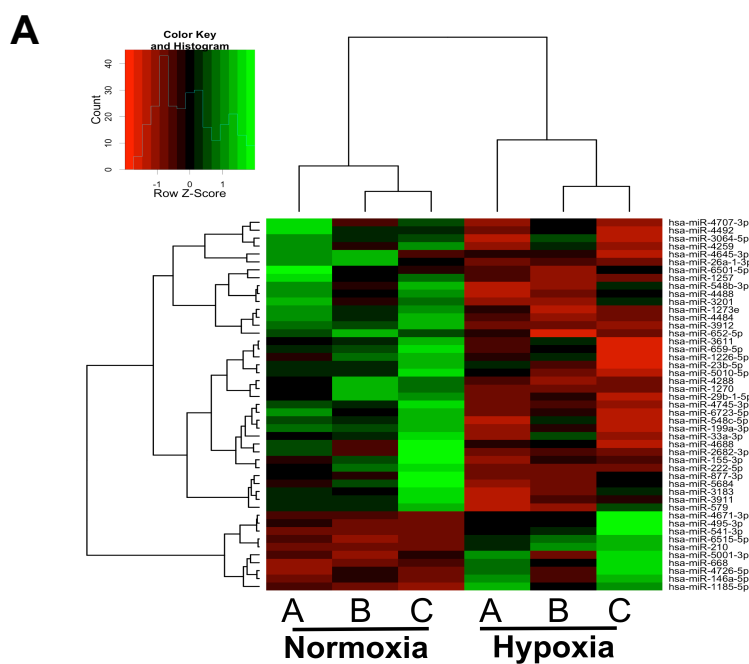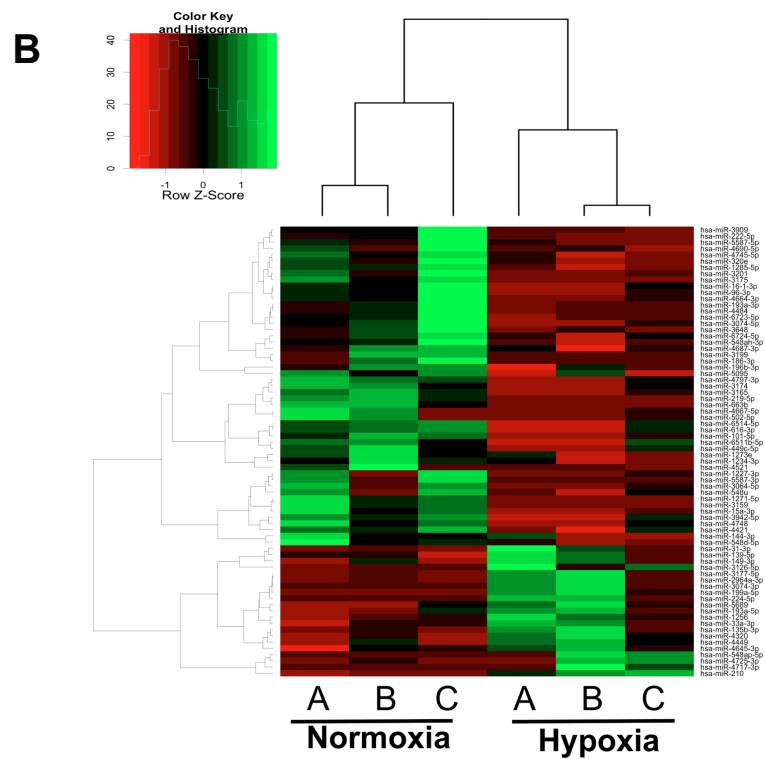

Supplement: S1 Fig — (A) Expression of hypoxamiRs in uninfected SLK cells. Heat map of miRNA changes between hypoxic and normoxic SLK cells in the six individual replicates (three normoxic and three hypoxic SLK samples). Presented is the relative expression of 47 significantly deregulated cellular miRNAs in hypoxic vs. normoxic infected cells (P-value ≤0.05, linear FC ≤-2 or ≥2) with an average read count ≥1. Using an uncentered Pearson correlation as the distance metric, an unsupervised hierarchical heatmap was generated, with each row represents a miRNA and each column representing a biological replicate. Red, black and green denote low, median and high relative miRNA expression, respectively. (B) Expression of hypoxamiRs in infected SLKK cells. Heat map of miRNA changes between hypoxic and normoxic SLKK cells in the six individual replicates (three normoxic and three hypoxic SLKK samples). Presented is the relative expression of 72 significantly deregulated cellular miRNAs in hypoxic vs. normoxic infected cells (P-value ≤0.05, linear FC ≤-2 or ≥2) with an average read count ≥1. The plot is depicted as in S1A Fig, with each row representing a miRNA and each column representing a biological replicate. Red, black and green denote low, median and high relative miRNA expression, respectively. (PDF) [file ppat.1006143.s001.pdf]

S2 Figure.

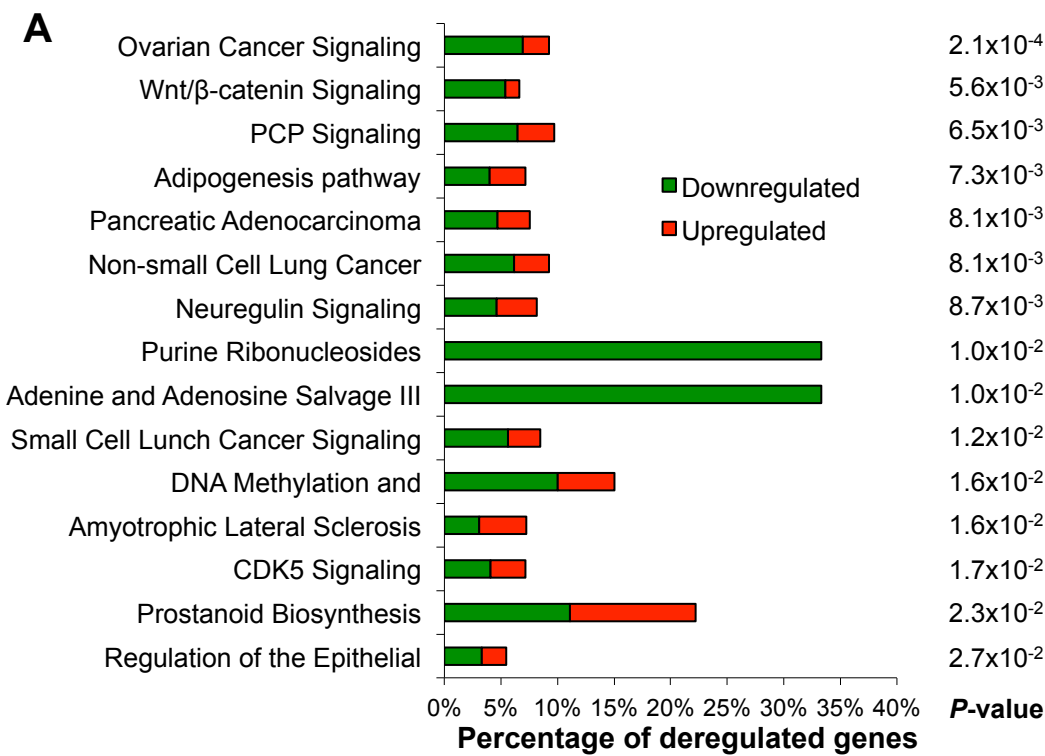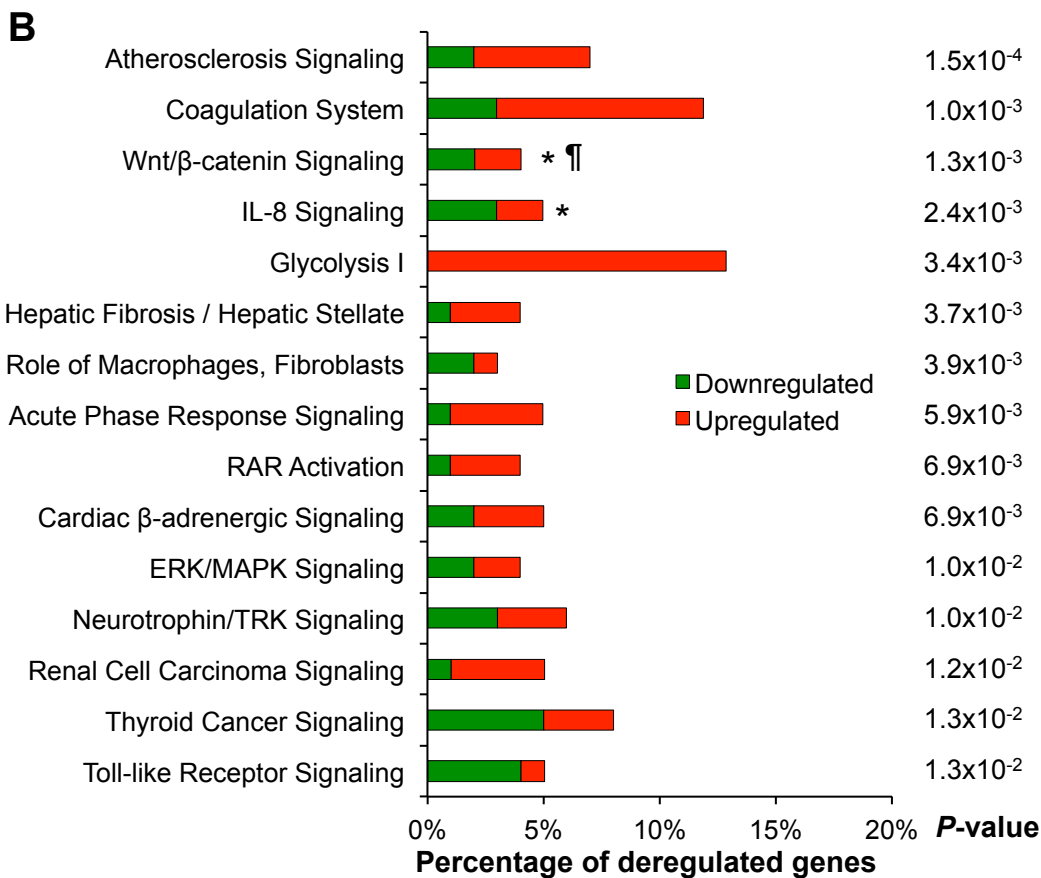

Supplement: S2 Fig — (A) Top 15 pathways altered by hypoxia in SLK cells. A list of differentially expressed mRNAs was used as input for Ingenuity Pathway Analysis, generating pathways altered by hypoxia in uninfected SLK cells. The 15 pathways that are most significantly altered are illustrated here. The yellow line reflects the significance (P ≤0.05 when–log(P-value) >1.3). The percentage of down-regulated and up-regulated genes in a given pathway is depicted in green and red, respectively. (B) Top 15 pathways altered by hypoxia in SLKK cells. A list of differentially expressed mRNAs in SLKK cells was used for Ingenuity Pathway Analysis. The 15 pathways that were most significantly altered by hypoxia are presented here. For any given pathway, the percentage of down-regulated and up-regulated genes are depicted in green and red, respectively. The total number of genes in each pathway is indicated in bold on the right hand side. An asterisk * indicates pathways that were also found altered due to infection alone [25]. The symbol (¶) indicates pathways that were also found altered by hypoxia in uninfected SLK cells. The significance of each pathways altered by hypoxia in SLKK cells was assessed by P-value. (PDF) [file ppat.1006143.s002.pdf]

**S4 Figure.**

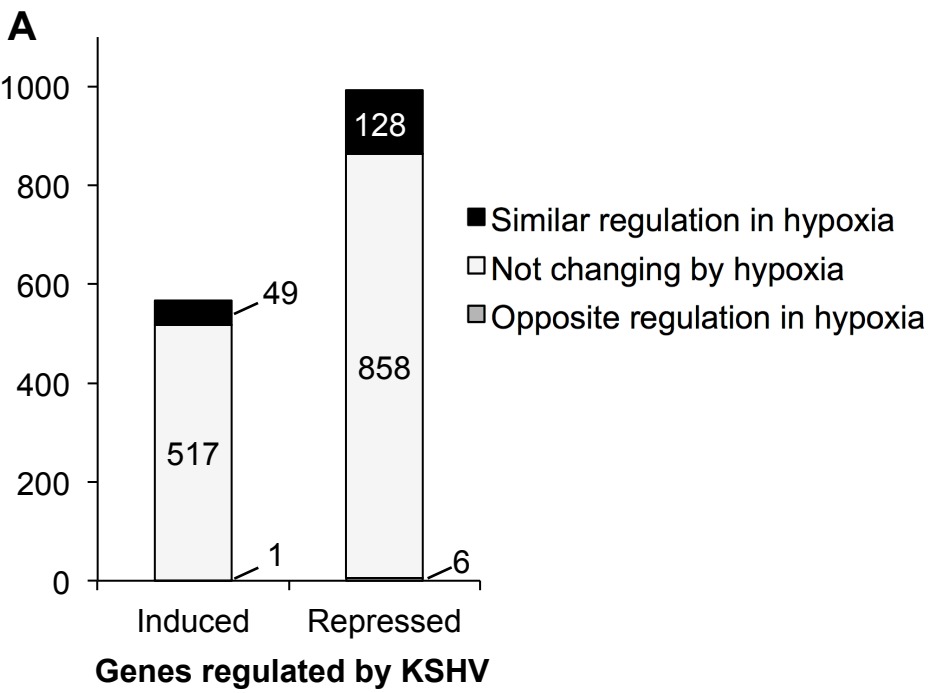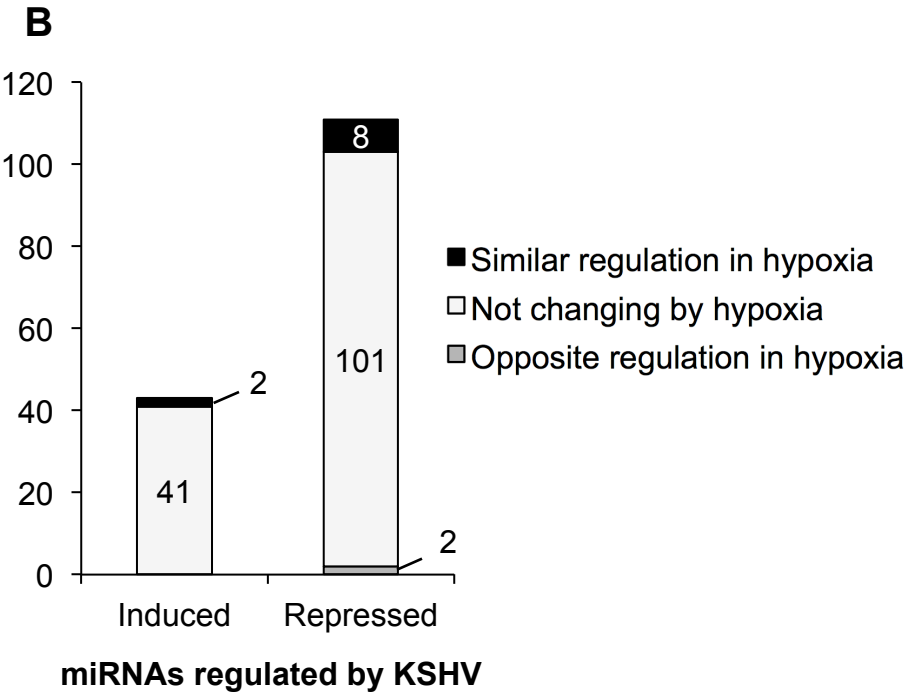

Supplement: S4 Fig — This illustrates to what extent genes (S4A) and miRNAs (S4B) that are regulated by KSHV infection [25] are also changing due to hypoxia. The analysis is done as in Fig 2A. (PDF) [file ppat.1006143.s004.pdf]

S5 Figure.

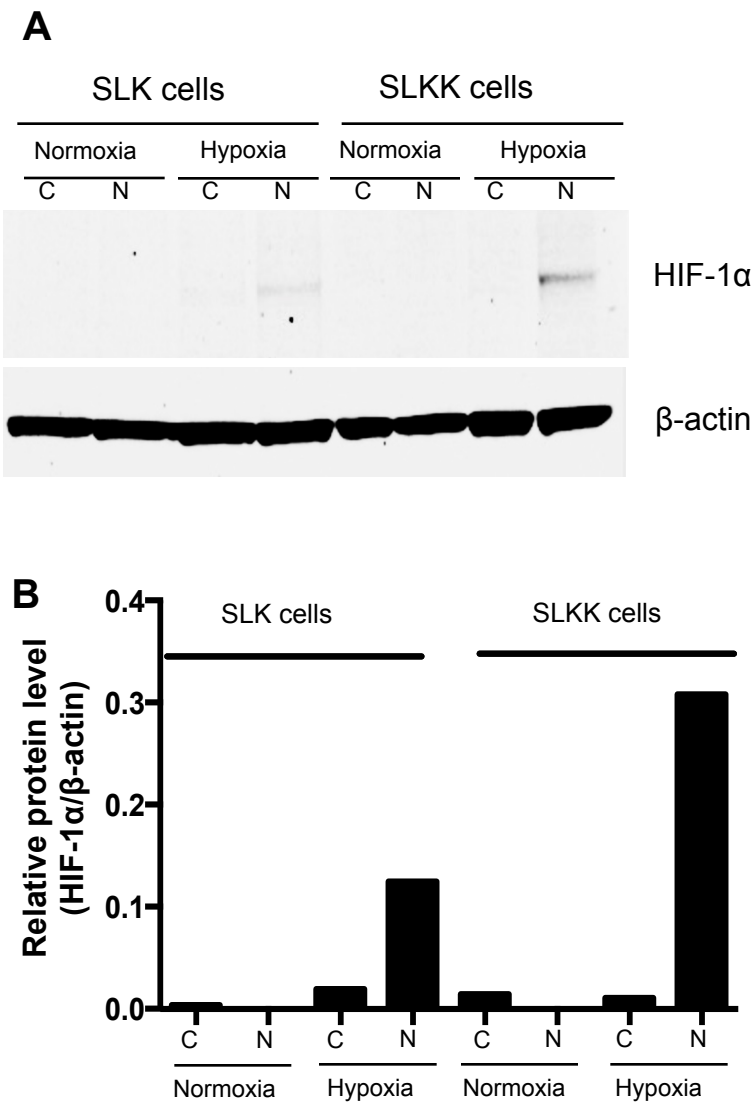

Supplement: S5 Fig — We determined the level of HIF-1α protein in the cytoplasmic and nuclear fractions of SLK and SLKK cells, under hypoxia and normoxia. C and N stand for cytoplasmic and nuclear fraction, respectively. S5A shows Western Blot analyses of HIF-1α, while S5B shows the intensities of immunoreactive bands quantified by densitometric analysis. (PDF) [file ppat.1006143.s005.pdf]

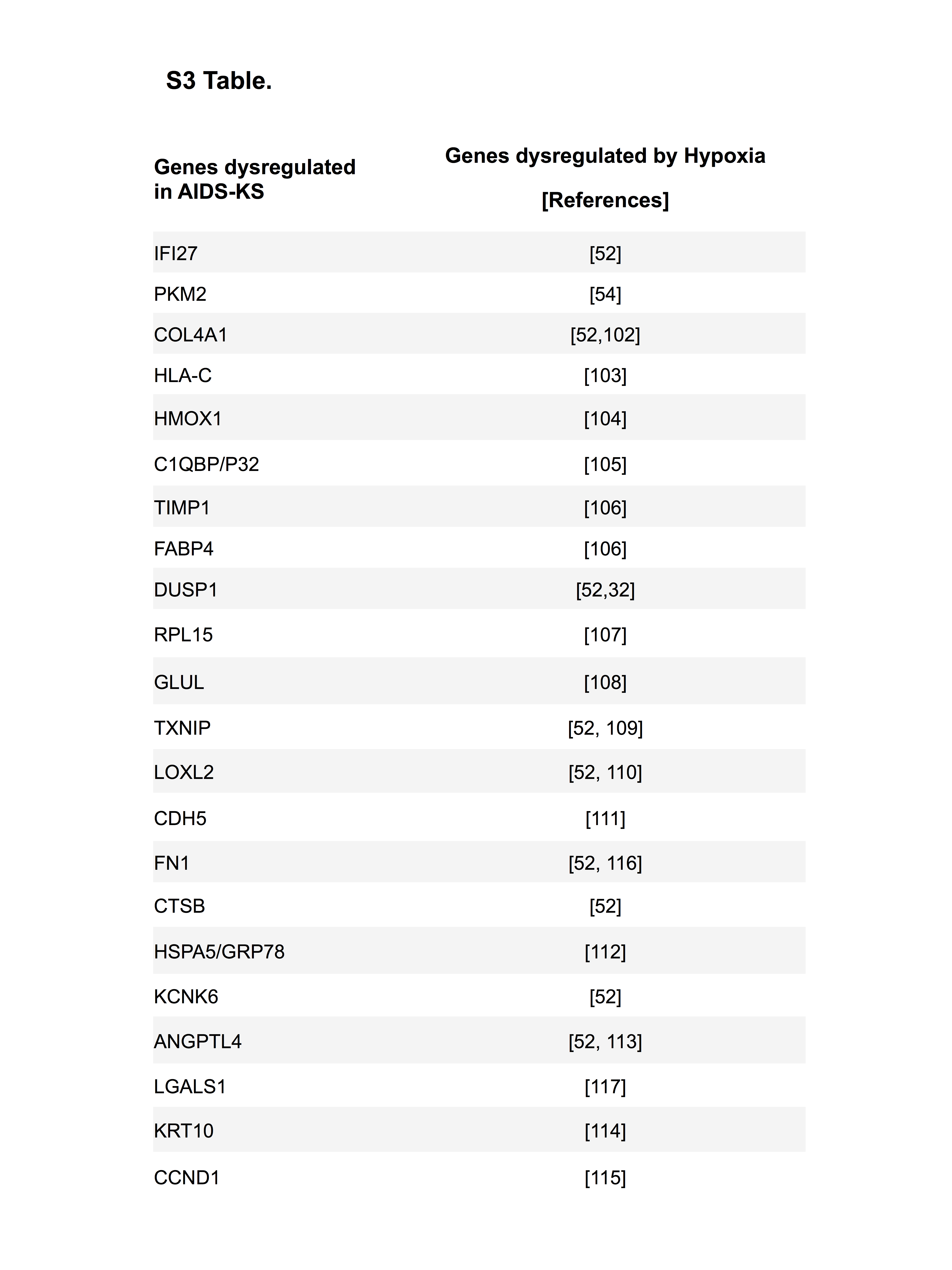

Supplement: S3 Table — These 22 genes are similarly regulated in AIDS-KS and in hypoxia. Hypoxic signatures from SLK cells, SLKK cells, HUVECs [52], as well as other cell lines [32,102–117] were compared to the 76 key host genes dysregulated in AIDS-KS [53]. Also, HMOX1, DUSP1 and LGALS1 were significantly induced by hypoxia in SLKK cells, and TXNIP was up-regulated in both SLK and SLKK hypoxic cells. (PNG) [file ppat.1006143.s008.png]
