# Supplementary material for: RNA Sequencing Reveals that Kaposi Sarcoma-Associated Herpesvirus Infection Mimics Hypoxia Gene Expression Signature
Source: PLoS Pathog. 2017 Jan 3;13(1):e1006143. doi: 10.1371/journal.ppat.1006143 (PMC5234848; doi:10.1371/journal.ppat.1006143)
Supplement: S3 Fig — (A) Workflow of integrated miRNA-mRNA association analysis using IPA. This experimental workflow shows the various filters used to associate miRNA-Seq and mRNA-Seq data from hypoxic and normoxic SLK cells. Numbers are presented as Total differentially expressed miRNAs or mRNAs (Up-regulated/Down-regulated). (B) Ingenuity analysis predicts inversely correlated miRNA-mRNA target pairs in hypoxic vs. normoxic uninfected SLK cells. Using lists of differentially expressed miRNAs and mRNAs as input for the Ingenuity Pathway Analysis (IPA), it was found that 35 differentially expressed miRNAs target 108 mRNAs (high confidence and experimentally observed results). Only mRNAs and miRNAs with inverse differential expression are shown in this table. Columns identify the miRNA name, its log-transformed expression fold change between hypoxic and normoxic SLK cells, the number of identified targets, and the five most differentially expressed targets. In bold are miRNAs and mRNA targets that have been previously reported in the literature. (PDF) [file ppat.1006143.s003.pdf]

S3 Figure.

A

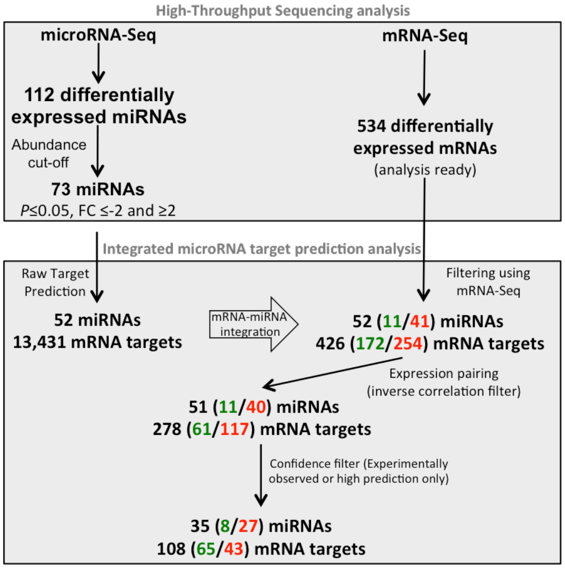

B

miRNA	Log_2 Fold Change	# Target genes	Top 5 target genes
**Up-regulated miRNAs/Down-regulated targets**			
miR-541-3p	7.9	15	BCL9L, MAST3, SPRYD4, HMGA1, VDR
**miR-210**	3.7	2	**HOXA1**, CLUH
miR-4671-3p	3.1	2	FOSL1, ZNF213
miR-4734	3.1	3	TMEM104, ANKRD52, CLUH
miR-4726-5p	2.2	10	SLC25A22, PYCRL, MAPKAPK3, PTGES, WNT7B
miR-495-3p	1.9	10	ZNF697, PSME3, MIDN, SOX9, GPR3
miR-1185-5p	1.7	2	BCAM, SH3RF2
**miR-146a-5p**	1.2	3	**PA2G4**, SMYD5, SSTR1
**Down-regulated miRNAs/Up-regulated targets**			
**miR-4458**	-7.5	20	**PTGS2**, YPEL2, CTHRC1, KLHL24, KLHL31
miR-548ar-3p	-7.2	2	PAG1, HPS2
miR-4793-3p	-7.0	2	ERRFI1, SLC38A2
miR-1270	-4.7	1	POLI
miR-548d-3p	-4.1	1	ZNF468
miR-663b	-3.9	4	SLC2A1, KDM7A, CLIC4, C1QL1
miR-4749-3p	-3.6	4	CD109, IPMK, KLF12, TNFAIP8
miR-1291	-3.5	1	PCMTD1
miR-4747-3p	-3.3	1	POLI
miR-4707-3p	-2.3	4	WSB1, PPP1R3B, KIAA1715, APOL1
miR-3130-3p	-2.0	1	SLC7A11
miR-2278	-1.9	2	PDP1, ZNF33B
miR-4467	-1.8	1	RTN4R
miR-1273e	-1.8	3	FBXL3, ZC3H6, ZNF468
miR-3201	-1.7	2	ITGA2, BNIP3L
miR-3911	-1.6	4	TES, IKBIP, EDN1, PTPRB
miR-3064-5p	-1.6	1	TXNIP
miR-4507	-1.6	2	ZBTB1, CCNG2
miR-4488	-1.5	3	GAL3ST1, NDRG1, C1QL1
miR-3136-5p	-1.5	4	SHOC2, ALG10B, IPMK, AHR
miR-4688	-1.4	2	CDK19, TBL1XR1
**miR-199a-3p**	-1.4	3	ITGB8, **PTGS2**, RNGTT
hsa-miR-5010-5p	-1.3	3	HK2, EDN1, STC1
hsa-miR-4492	-1.3	5	PJA2, TBL1XR1, PFKFB4, ZNF395, C1QL1
hsa-miR-766-3p	-1.3	3	TGFBR1, ZNF468, ERRFI1
hsa-miR-3175	-1.2	2	SLC2A1, AK4
hsa-miR-1257	-1.0	1	ZNF468
