# Supplementary material for: RNA Sequencing Reveals that Kaposi Sarcoma-Associated Herpesvirus Infection Mimics Hypoxia Gene Expression Signature
Source: PLoS Pathog. 2017 Jan 3;13(1):e1006143. doi: 10.1371/journal.ppat.1006143 (PMC5234848; doi:10.1371/journal.ppat.1006143)
Supplement: S1 Table — These 49 genes are up-regulated by both hypoxia and KSHV infection. The 5 most abundant mRNAs (average read count) appear in bold (DDIT4, IGFBP3, CLIC4, RSPS27L and NEAT1). (PDF) [file ppat.1006143.s006.pdf]

S1 Table.

**Gene**	**$SLK_{Hypoxia}$ vs. $SLK_{Normoxia}$**		**SLKK vs. SLK**		**Average Read count**
	Log_2 FC	P-value	Log_2 FC	P-value	
**DDIT4**	3.8	2.08E-16	1.2	1.80E-03	116.9
ARRDC3	5.4	2.22E-16	2.4	7.05E-06	7.7
STC2	3.9	2.24E-09	1.4	2.49E-03	1.1
PLOD2	3.0	1.44E-06	2.6	1.70E-07	31.1
**IGFBP3**	2.3	2.15E-06	1.6	2.84E-05	156.5
CCNG2	2.7	7.32E-06	1.3	4.13E-03	2.4
**CLIC4**	2.1	1.62E-05	1.8	1.03E-06	52.5
KLHL24	2.6	2.26E-05	1.4	4.54E-04	1.2
SLC38A2	1.8	2.28E-04	1.1	2.06E-03	31.8
VAV3	1.9	2.37E-03	2.1	1.19E-06	5.0
SLC25A36	1.8	2.52E-03	1.4	9.44E-04	7.0
BBS10	1.8	2.77E-03	1.4	1.91E-03	3.4
ADD3	1.5	2.84E-03	1.1	4.39E-03	14.7
NEK7	1.6	5.38E-03	1.6	7.71E-05	10.0
BIRC3	1.7	5.39E-03	3.9	1.33E-15	1.9
ZNF277	1.5	5.50E-03	1.4	7.34E-04	6.9
**RPS27L**	1.3	8.47E-03	1.4	1.99E-04	77.4
NR3C1	1.7	9.00E-03	1.4	1.12E-03	9.5
CD46	1.5	1.09E-02	1.7	5.93E-05	49.2
RNF19A	1.5	1.24E-02	1.3	3.79E-03	6.7
JHDM1D	1.5	1.25E-02	1.5	1.07E-04	1.0
CAPZA2	1.2	1.39E-02	1.3	8.42E-04	36.7
ZBTB1	1.5	1.39E-02	1.6	4.27E-04	5.9
ITGAV	1.3	1.54E-02	1.3	8.90E-04	25.3
TBL1XR1	1.5	1.73E-02	1.8	1.05E-04	13.5
DSG2	1.1	1.84E-02	1.5	2.49E-05	26.0
FGD4	1.5	2.16E-02	1.2	3.44E-03	0.5
OPTN	1.3	2.22E-02	1.3	1.14E-03	12.0
SNX6	1.1	2.26E-02	1.5	3.46E-05	27.4
TBC1D23	1.3	2.35E-02	1.4	6.96E-04	6.5
**NEAT1**	1.1	2.69E-02	3.0	8.75E-14	86.7
KLHL9	1.3	2.79E-02	1.2	3.78E-03	3.9
RSL24D1	1.1	2.80E-02	2.2	1.32E-08	47.2
PJA2	1.1	2.86E-02	1.4	3.81E-04	11.1
GALNT3	1.3	3.13E-02	2.3	7.74E-08	5.4
TRIM59	1.1	3.14E-02	1.3	1.04E-03	10.0
ACTR6	1.2	3.15E-02	1.4	1.26E-03	7.3
CD109	1.2	3.22E-02	1.4	8.58E-04	3.7
PAIP2	1.1	3.38E-02	1.4	5.90E-04	56.7
PPP1CB	1.2	3.43E-02	1.3	1.91E-03	27.1
NDRG1	2.4	3.47E-02	2.6	2.46E-04	0.3
PDP1	1.2	3.69E-02	1.4	2.09E-03	5.8
FBXL3	1.3	3.90E-02	1.5	5.16E-04	9.5
TRAPPC8	1.2	4.12E-02	1.6	4.73E-04	3.7
C12orf23	1.1	4.33E-02	1.3	1.33E-03	21.1
TWSG1	1.0	4.37E-02	1.4	2.76E-04	16.1
C1orf96	1.1	4.38E-02	1.1	3.44E-03	4.7
NUF2	1.2	4.58E-02	1.2	3.91E-03	24.2
SLC7A11	1.1	4.73E-02	2.2	1.44E-07	1.7
