# Supplementary material for: RNA Sequencing Reveals that Kaposi Sarcoma-Associated Herpesvirus Infection Mimics Hypoxia Gene Expression Signature
Source: PLoS Pathog. 2017 Jan 3;13(1):e1006143. doi: 10.1371/journal.ppat.1006143 (PMC5234848; doi:10.1371/journal.ppat.1006143)
Supplement: S2 Table — These 40 genes are up-regulated by both hypoxia and KSHV de novo infection (48 hrs post infection) in HUVECs [51,52]. NDRG1, which was also found up-regulated in the SLK/SLKK cell model, is in bold. (PDF) [file ppat.1006143.s007.pdf]

S2 Table.

Gene	Fold change $HUVEC_{Hypoxia}$ vs. $HUVEC_{Normoxia}$	Fold Change $HUVEC_{kshv}$ vs. $HUVEC_{mock}$
**NDRG1**	4.9	1.9
C10orf10	4.9	2.4
FBXO32	4.5	1.9
C1orf21	3.9	2.0
PLAC8	3.9	2.5
JUNB	3.8	1.8
MMP11	3.8	1.5
IFITM1	3.6	2.1
SULF2	3.5	2.3
ARL4C	3.2	2.1
CNKSR3	3.2	2.2
CTHRC1	2.8	1.6
JAK3	2.8	2.6
IFI27	2.8	2.0
CADM1	2.7	1.7
CSGALNACT1	2.6	2.0
APOLD1	2.5	1.7
TTLL3	2.4	2.3
ITM2A	2.4	1.5
CASP1	2.4	2.0
ST3GAL6	2.4	1.5
ARID5A	2.4	1.8
CDH13	2.3	1.9
TCEA3	2.3	2.1
ACYP2	2.3	1.5
UACA	2.2	1.8
NEDD4L	2.1	2.1
DUSP1	2.1	1.8
CTSS	2.1	2.2
GMFG	2.1	2.2
SPRY1	2.1	2.1
PTPRM	2.0	1.5
PROS1	1.9	1.7
PDE2A	1.9	1.5
DIAPH2	1.9	1.8
CLEC2B	1.9	2.1
ITM2B	1.6	1.6
SERPINB1	1.6	1.8
LPAR6	1.5	1.6
CD55	1.5	3.7
