# Supplementary material for: RNA Sequencing Reveals that Kaposi Sarcoma-Associated Herpesvirus Infection Mimics Hypoxia Gene Expression Signature
Source: PLoS Pathog. 2017 Jan 3;13(1):e1006143. doi: 10.1371/journal.ppat.1006143 (PMC5234848; doi:10.1371/journal.ppat.1006143)
Supplement: S4 Table — Three independent experiments are displayed (A, B, and C). Columns identify the replicate number, the number of aligned miRNA reads per species and total, and the percentage of KSHV miRNA reads vs. the total number of aligned reads for each SLKK replicate and overall. (PDF) [file ppat.1006143.s009.pdf]

S4 Table.

SLKK	Aligned miRNA reads			Percentage of aligned KSHV miRNA reads vs. Total
	Total	Human	KSHV	
Normoxia A	1,101,677	954,226	147,451	13.4%
Normoxia B	1,684,493	1,558,250	126,243	7.5%
Normoxia C	691,718	641,567	50,151	7.3%
**Average Normoxia**	**1,159,296**	**1,051,348**	**107,948**	**9.4%**
Hypoxia A	3,232,670	2,955,249	277,421	8.6%
Hypoxia B	661,448	575,814	85,634	12.3%
Hypoxia C	460,587	425,181	35,406	7.7%
**Average Hypoxia**	**1,451,568**	**1,318,748**	**132,820**	**9.7%**
