# Supplementary material for: RNA Sequencing Reveals that Kaposi Sarcoma-Associated Herpesvirus Infection Mimics Hypoxia Gene Expression Signature
Source: PLoS Pathog. 2017 Jan 3;13(1):e1006143. doi: 10.1371/journal.ppat.1006143 (PMC5234848; doi:10.1371/journal.ppat.1006143)
Supplement: S5 Table — The average of three independent experiments for each condition is displayed. Columns identify each KSHV miRNA, its total miR count, its percentage as compared to either KSHV miR reads or the overall read number, in either normoxia or hypoxia. The top part illustrates KSHV miRNAs present in more than 1% of total KSHV reads. The rest is displayed under “Others”. KSHV miRNAs in bold have been validated by Taqman assays (see Fig 3G). (PDF) [file ppat.1006143.s010.pdf]

S5 Table.

KSHV miRNA	Normoxic SLKK cells			Hypoxic SLKK cells		
	**Count**	**Percentage of Total**	**Percentage of KSHV**	**Count**	**Percentage of Total**	**Percentage of KSHV**
**kshv-miR-K12-10a-3p**	405,293	3.81	40.53	410,783	3.98	41.08
**kshv-miR-K12-4-3p**	261,969	2.46	26.20	256,130	2.48	25.61
**kshv-miR-K12-8-3p**	126,885	1.19	12.69	106,622	1.03	10.66
**kshv-miR-K12-3-5p**	51,448	0.48	6.05	51,753	0.50	5.18
kshv-miR-K12-10b	46,503	0.44	4.65	60,362	0.59	6.04
kshv-miR-K12-7-3p	18,871	0.18	1.89	17,393	0.17	1.74
**kshv-miR-K12-2-5p**	17,029	0.16	1.70	14,982	0.15	1.50
**kshv-miR-K12-6-3p**	15,408	0.14	1.54	16,016	0.16	1.60
**kshv-miR-K12-11-3p**	14,417	0.14	1.44	18,543	0.18	1.85
kshv-miR-K12-12-5p	11,199	0.11	1.12	17,288	0.17	1.73
kshv-miR-K12-4-5p	10,389	0.10	1.04	8,259	0.08	0.83
Others	20,588	0.19	2.06	21,871	0.21	2.19
kshv-miR-K12-6-5p	5,124	0.05	0.51	7,135	0.07	0.71
kshv-miR-K12-3-3p	3,251	0.03	0.33	1,527	0.01	0.15
kshv-miR-K12-12-3p	2,938	0.03	0.29	4,359	0.04	0.44
**kshv-miR-K12-1-5p**	2,789	0.03	0.28	1,820	0.02	0.18
kshv-miR-K12-5-3p	2,657	0.02	0.27	2,787	0.03	0.28
kshv-miR-K12-9-3p	1,568	0.01	0.16	1,257	0.01	0.13
kshv-miR-K12-8-5p	962	0.01	0.10	1,521	0.01	0.15
kshv-miR-K12-9-5p	562	0.01	0.06	772	0.01	0.08
kshv-miR-K12-7-5p	401	0.00	0.04	254	0.00	0.03
kshv-miR-K12-2-3p	182	0.00	0.02	197	0.00	0.02
kshv-miR-K12-10a-5p	139	0.00	0.01	218	0.00	0.02
kshv-miR-K12-1-3p	7	0.00	0.00	10	0.00	0.00
kshv-miR-K12-11-5p	5	0.00	0.00	1	0.00	0.00
kshv-miR-K12-5-5p	3	0.00	0.00	12	0.00	0.00
